# Structural and Mechanistic Insight into the *Listeria monocytogenes* Two-enzyme Lipoteichoic Acid Synthesis System*[Fn FN2]

**DOI:** 10.1074/jbc.M114.590570

**Published:** 2014-08-15

**Authors:** Ivan Campeotto, Matthew G. Percy, James T. MacDonald, Andreas Förster, Paul S. Freemont, Angelika Gründling

**Affiliations:** From the ‡ Section of Microbiology and MRC Centre for Molecular Bacteriology and Infection, and; the § Centre for Structural Biology, Imperial College London, London SW7 2AZ, United Kingdom

**Keywords:** Bacteria, Cell Wall, Enzyme Catalysis, Lipid, Protein Structure, LTA Synthesis

## Abstract

Lipoteichoic acid (LTA) is an important cell wall component required for proper cell growth in many Gram-positive bacteria. In *Listeria monocytogenes*, two enzymes are required for the synthesis of this polyglycerolphosphate polymer. The LTA primase LtaP_Lm_ initiates LTA synthesis by transferring the first glycerolphosphate (GroP) subunit onto the glycolipid anchor and the LTA synthase LtaS_Lm_ extends the polymer by the repeated addition of GroP subunits to the tip of the growing chain. Here, we present the crystal structures of the enzymatic domains of LtaP_Lm_ and LtaS_Lm_. Although the enzymes share the same fold, substantial differences in the cavity of the catalytic site and surface charge distribution contribute to enzyme specialization. The eLtaS_Lm_ structure was also determined in complex with GroP revealing a second GroP binding site. Mutational analysis confirmed an essential function for this binding site and allowed us to propose a model for the binding of the growing chain.

## Teaser

**Background:** *Listeria monocytogenes* lipoteichoic acid is synthesized by the LtaP/LtaS two-enzyme system.

**Results:** Structural analysis reveals a second glycerolphosphate binding site in LtaS important for *in vitro* and *in vivo* enzyme function.

**Conclusion:** These results suggest a binding mode for the lipoteichoic acid chain during polymerization.

**Significance:** The identified binding site in LtaS could become a target for antibiotic development.

## Introduction

Lipoteichoic acid (LTA)^3^ is an important cell wall component found in many Gram-positive bacteria, including human pathogens such as *Staphylococcus aureus* and *Listeria monocytogenes*. In its absence, bacteria are impaired in growth and show cell morphology and cell division defects (1–3). Therefore, enzymes involved in its synthesis are attractive targets for the design of new antimicrobials. This has been experimentally validated with the identification of a small molecule LTA synthesis inhibitor that prevented the growth of antibiotic-resistant Gram-positive bacteria as well as prolonging the survival of mice challenged with a lethal dose of *S. aureus* (4).

A common type of LTA consists of a linear 1,3-linked polyglycerolphosphate (PGP) polymer that is attached to the outside of the membrane via a glycolipid anchor (5, 6). In *L. monocytogenes*, the glycolipids anchor is Gal(α1–2)-Glc(α1–3)-diacylglycerol (Gal-Glc-DAG) or Gal(α1–2)Ptd-6-Glc(α1–3)-DAG (Gal-Ptd-6Glc-DAG), in which the glucose moiety is lipidated with an additional phosphatidyl (Ptd) group (5, 7, 8). The PGP backbone chain is polymerized by lipoteichoic acid synthase or LtaS-type enzymes (1). This class of enzyme uses the membrane lipid phosphatidylglycerolphosphate (PG) as a substrate, hydrolyzes the glycerolphosphate (GroP) head group of this lipid and subsequently adds it to the tip of the growing chain (9, 10). In *S. aureus* only one enzyme, namely LtaS_Sa_, is required for LTA backbone synthesis. This enzyme initiates LTA synthesis by the transfer of the first GroP subunit onto the glycolipid anchor and subsequently polymerizes the backbone chain by the repeated addition of GroP subunits (1, 11). In contrast, *L. monocytogenes* uses a two-enzyme system for LTA synthesis (3). The lipoteichoic acid primase LtaP_Lm_ transfers the initial GroP subunits to the glycolipid anchor but is unable to extend the chain further. Chain polymerization is performed by the lipoteichoic acid synthase LtaS_Lm_ (3).

Regardless of whether LTA synthase or primase, LtaS-type enzymes, have the same overall architecture. They are composed of an N-terminal domain with five transmembrane helices, which is followed by an extracellular C-terminal domain (eLtaS) containing the catalytic site (recently reviewed in Ref. 12). For many organisms, including the human pathogens *S. aureus, Staphylococcus epidermidis*, *L. monocytogenes*, and *Bacillus anthracis*, it has been shown that LtaS is cleaved by an endogenous peptidase and a fraction of the extracellular eLtaS is released into the culture supernatant as well as partially retained within the cell wall fraction (3, 13–16). *In vitro*, the extracellular eLtaS has been shown to be sufficient for PG hydrolysis (11, 17). However, expression of the extracellular enzymatic domain is not sufficient for LTA production *in vivo* and the full-length membrane embedded LtaS protein is required for polymer production (16).

The structures of the extracellular enzymatic eLtaS domains of the *S. aureus* (PDB code 2W5Q) and *B. subtilis* (PDB code 2W8D) have been reported (13, 18). These previous studies showed that the enzymes are related to arylsulfatase family enzymes with the same α/β-barrel fold. A conserved metal binding site was revealed and its requirement for enzyme function confirmed experimentally (13). In addition, a Thr amino acid within the active center was identified as the catalytic residue and its essential role was confirmed as an LtaS_Sa_-T300A variant was enzymatically inactive both *in vitro* and *in vivo* (13). The active site Thr was found to be phosphorylated in the *B. subtilis* and unmodified in the *S. aureus* structure, but the biological significance of this modification has not yet been determined. It was further hypothesized that the reaction proceeds through a covalent GroP-enzyme intermediate through the catalytic Thr (13).

To understand better the reaction mechanism and enzyme specificity of this class of proteins, we performed a structural analysis of the extracellular soluble domains of the two *L. monocytogenes* enzymes eLtaP_Lm_ and eLtaS_Lm_. This analysis revealed a substantially smaller cavity around the catalytic center in the primase enzyme compared with the synthase enzyme. The eLtaS_Lm_ structure was also determined in complex with GroP. This led to the identification of a second GroP binding site in eLtaS_Lm_ that is essential for enzyme function. Detailed bioinformatics analyses revealed specific motifs that differentiate LtaS and LtaP enzymes and highlighted that primase-related enzymes are only present in a small subset of bacteria. Taken together the structural and functional data allowed us to propose a revised mechanism for LTA biosynthesis in Gram-positive bacteria.

## EXPERIMENTAL PROCEDURES

### 

#### 

##### Plasmid and Strain Construction

Strains and primers used in this study are listed in Tables 1 and 2, respectively. *Escherichia coli* strains were grown in LB medium and *L. monocytogenes* strains in BHI medium. The cultures were grown at the indicated temperatures and the growth medium was supplemented with antibiotics as indicated in Table 1. Plasmids for the expression of eLtaS_Lm_ variants with T307A, S486A, N488A, and H489A single amino acid substitutions and the triple mutant S486A/N488A/H489A (AAA variant) were constructed by QuikChange mutagenesis using plasmid pProEX-eLtaS_Lm_ (strain ANG1449) as template and primer pairs ANG1649/ANG1650, ANG1651/ANG1652, ANG1653/ANG1654, ANG1655/ANG1656, and ANG1657/ANG1658. The resulting plasmids were initially transformed into *E. coli* strain XL1-Blue yielding strains ANG2935 to ANG2939 and subsequently transformed for protein expressing into the *E. coli* Rosetta strain yielding strains ANG2940 to ANG2944. Plasmid pPL3-*lmo0927His6* (Strain ANG1401) allows for the expression of full-length LtaS_Lm_ with a C-terminal His tag from its native promoter in *L. monocytogenes* (3). This vector was used as template for the construction of plasmids pPL3-*lmo0927His6-*T307A, pPL3-*lmo0927His6-*S286A, pPL3-*lmo0927His6-*N488A, pPL3-*lmo0927His6-*H489A, pPL3-*lmo0927His6-*AAA for the expression of the different LtaS_Lm_ variants in *L. monocytogenes.* The desired mutations were introduced by SOE PCR. More specifically, plasmid pPL3-*lmo0927His6-*T307A was constructed by amplifying the front and back of *lmo0927* and introducing the desired point mutation using plasmid pPL3-*lmo0927His6* as template and primer pairs ANG674/ANG1650 and ANG676/ANG1649 in two separate PCR reactions. The two fragments were subsequently fused in a second round of PCR using primers ANG674/ANG676. The resulting product was digested with PstI and SalI and ligated with vector pPL3 that has been cut with the same enzymes. Plasmids pPL3-*lmo0927His6-*S286A, pPL3-*lmo0927His6-*N488A, pPL3-*lmo0927His6-*H489A, and pPL3-*lmo0927His6-*AAA were constructed using the same strategy and primers ANG1652 to ANG1658 as listed in Table 2. The resulting plasmids were initially recovered in *E. coli* strain XL1-Blue yielding strains ANG2930 to ANG2934 and subsequently transformed along with plasmid pPL3-*lmo0927His6* into *E. coli* strain SM10 yielding strains ANG1460 and ANG2946 to ANG2950. Next all plasmids were conjugated from SM10 into *L. monocytogenes* strain 10403SΔ*lmo0927* using a previously described method (19) but maintaining the *L. monocytogenes* 10403SΔ*lmo0927* strain at 30 °C throughout the procedure. This yielded *L. monocytogenes* strains ANG1454, and ANG2951 to ANG2955, which were also propagated at 30 °C. The sequences of all inserts were verified by automated fluorescence sequencing at the MRC Clinical Sciences Centre Genomics Core Laboratory, Imperial College London.

**TABLE 1:** **Bacterial strains used in this study** Antibiotics were used at the following concentrations: for *E. coli* cultures: Ampicillin (AmpR) 100 μg/ml; kanamycin (KanR), 30 μg/ml; tetracycline (TetR), 10 μg/ml; for *L. monocytogenes* cultures: chloramphenicol (CamR), 7.5 μg/ml; streptomycin, 200 μg/ml (StrepR) for conjugation experiments.

Strain	Relevant features	Reference
***Escherichia coli* strains**		
XL1 Blue	Cloning strain, TetR – ANG127	Stratagene
SM10	*E. coli* strain used for conjugations; KanR – ANG618	40
DH-E898	XL1 Blue pPL3; *L. monocytogenes* integration vector; CamR – ANG1276	41
ANG1401	XL1 Blue pPL3-*lmo0927His6*; Lmo0927 (LtaS_Lm_) with C-terminal His-tag under native promoter control; CamR	3
ANG1449	DH5α pProEX-eLtaS_Lm_; plasmid for expression of eLtaS_Lm_; AmpR	11
ANG1478	Rosetta pProEX-eLtaP_Lm_; strain for overexpression of eLtaP_Lm_; AmpR	11
ANG1479	Rosetta pProEX-eLtaS_Lm_; strain for overexpression of eLtaS_Lm_; AmpR	11
ANG2930	XL1-Blue pPL3-*lmo0927His6*-T307A; Lmo0927-T307A with C-terminal His-tag under native promoter control; CamR	This study
ANG2931	XL1-Blue pPL3-*lmo0927His6*-S486A; Lmo0927-S486A with C-terminal His-tag under native promoter control; CamR	This study
ANG2932	XL1-Blue pPL3-*lmo0927His6*-N488A; Lmo0927-N488A with C-terminal His-tag under native promoter control; CamR	This study
ANG2933	XL1-Blue pPL3-*lmo0927His6*-H489A; Lmo0927-H489A with C-terminal His-tag under native promoter control; CamR	This study
ANG2934	XL1-Blue pPL3-*lmo0927His6*-AAA; Lmo0927-AAA with C-terminal His-tag under native promoter control; CamR	This study
ANG2935	XL1-Blue pProEX-eLtaS_Lm_-T307A; plasmid for expression of eLtaS_Lm_-T307A variant; AmpR	This study
ANG2936	XL1-Blue pProEX-eLtaS_Lm_-S486A; plasmid for expression of eLtaS_Lm_-S486A variant; AmpR	This study
ANG2937	XL1-Blue pProEX-eLtaS_Lm_-N488A; plasmid for expression of eLtaS_Lm_-N488A variant; AmpR	This study
ANG2938	XL1-Blue pProEX-eLtaS_Lm_-H489A; plasmid for expression of eLtaS_Lm_-H489A variant; AmpR	This study
ANG2939	XL1-Blue pProEX-eLtaS_Lm_-AAA; plasmid for expression of eLtaS_Lm_-AAA variant; AmpR	This study
ANG2940	Rosetta pProEX-eLtaS_Lm_-T307A; strain for overexpression of eLtaS_Lm_-T307A variant; AmpR	This study
ANG2941	Rosetta pProEX-eLtaS_Lm_-S486A; strain for overexpression of eLtaS_Lm_-S486A variant; AmpR	This study
ANG2942	Rosetta pProEX-eLtaS_Lm_-N488A; strain for overexpression of eLtaS_Lm_-N488A variant; AmpR	This study
ANG2943	Rosetta pProEX-eLtaS_Lm_-H489A; strain for overexpression of eLtaS_Lm_-H489A variant; AmpR	This study
ANG2944	Rosetta pProEX-eLtaS_Lm_-AAA; strain for overexpression of eLtaS_Lm_-AAA variant; AmpR	This study
ANG1460	SM10 pPL3-*lmo0927His6*; KanR, CamR	This study
ANG2946	SM10 pPL3-*lmo0927His6*-T307A; KanR, CamR	This study
ANG2947	SM10 pPL3-*lmo0927His6*-S486A; KanR, CamR	This study
ANG2948	SM10 pPL3-*lmo0927His6*-N488A; KanR, CamR	This study
ANG2949	SM10 pPL3-*lmo0927His6*-H489A; KanR, CamR	This study
ANG2950	SM10 pPL3-*lmo0927His6*-AAA; KanR, CamR	This study

***Listeria monocytogenes* strains**		
10403S	StrepR – ANG1263	42
ANG1386	10403SΔ*lmo0927*; StrepR	This study
ANG1411	10403SΔ*lmo0927* pPL3; StrepR, CamR	This study
ANG1454	10403SΔ*lmo0927* pPL3-*lmo0927His6*; StrepR, CamR	This study
ANG2951	10403SΔ*lmo0927* pPL3-*lmo0927His6*-T307A; StrepR, CamR	This study
ANG2952	10403SΔ*lmo0927* pPL3-*lmo0927His6*-S486A; StrepR, CamR	This study
ANG2953	10403SΔ*lmo0927* pPL3-*lmo0927His6*-N488A; StrepR, CamR	This study
ANG2954	10403SΔ*lmo0927* pPL3-*lmo0927His6*-H489A; StrepR, CamR	This study
ANG2955	10403SΔ*lmo0927* pPL3-*lmo0927His6*-AAA; StrepR, CamR	This study

**TABLE 2:** **Primers used in this study** Restriction sites are underlined.

Number	Name	Sequence
ANG674	5-PstI-Lmo0927-withP	AACTGCAGCTAGCAGACTTCCATTCCAAATGGTTC
ANG676	3-SalI-Lmo0927-C-His	ACGCGTCGACTTAGTGATGGTGATGGTGATGaccTTTATCGGATGAATCAGTTGATTTTTTC
ANG1649	5-Lmo0927-T307A	CCACCAAACTGGACAAGGGAAAGCAGCTGACTCCGAAATGTTAC
ANG1650	3-Lmo0927-T307A	GTAACATTTCGGAGTCAGCTGCTTTCCCTTGTCCAGTTTGGTGG
ANG1651	5-Lmo0927-S486A	GTACGGTGACCATTATGGTATTGCCGACAACCATGAAGAAGCAATG
ANG1652	3-Lmo0927-S486A	CATTGCTTCTTCATGGTTGTCGGCAATACCATAATGGTCACCGTAC
ANG1653	5-Lmo0927-N488A	GACCATTATGGTATTTCCGACGCCCATGAAGAAGCAATGACAAAAATTC
ANG1654	3-Lmo0927-N488A	GAATTTTTGTCATTGCTTCTTCATGGGCGTCGGAAATACCATAATGGTC
ANG1655	5-Lmo0927-H489A	CCATTATGGTATTTCCGACAACGCTGAAGAAGCAATGACAAAAATTCTTG
ANG1656	3-Lmo0927-H489A	CAAGAATTTTTGTCATTGCTTCTTCAGCGTTGTCGGAAATACCATAATGG
ANG1657	5-Lmo0927-AAA	GTACGGTGACCATTATGGTATTGCCGACGCCGCTGAAGAAGCAATGACAAAAATTCTTG
ANG1658	3-Lmo0927-AAA	CAAGAATTTTTGTCATTGCTTCTTCAGCGGCGTCGGCAATACCATAATGGTCACCGTAC

##### Protein Expression and Purification

Strains ANG1478 Rosetta pProEX-eLtaP_Lm_ (11) and ANG1479 Rosetta pProEX-eLtaS_Lm_ (11) were used for the expression and purification of N terminally His-tagged eLtaP_Lm_ and eLtaS_Lm_ proteins, respectively. Protein induction and nickel affinity purification were performed as previously described (11, 13). The proteins were further purified by size exclusion chromatography using a Superdex S200 16/60 column (GE Healthcare) and a 50 mm Tris-HCl, pH 7.5, 200 mm NaCl, 5% glycerol buffer system for eLtaS_Lm_ and the different alanine substitution variants or 20 mm Tris-HCl, pH 7.5, for eLtaP_Lm_. Protein-containing fractions spanning the main peak were pooled and concentrated to ∼10 mg/ml using 10-kDa molecular mass cut-off Amicon filtration devices (Millipore), if not otherwise stated. These proteins were subsequently used in structural studies. eLtaS variants with T307A, S486A, N488A, and H489A single amino acid substitutions and a S486A/N488A/H489A (AAA variant) triple mutant were expressed in *E. coli* strains ANG2940 to ANG2944 (Table 1). *L. monocytogenes* strain 10403S pPL3-LtaS_Lm_-His_6_ (ANG1424) (3) was used for the expression and purification of eLtaS_Lm_ from the native host.

##### Purification of Native eLtaS_Lm_ from L. monocytogenes Culture Supernatant and Mass Spectrometry Analysis

The *L. monocytogenes* strain 10403S pPL3-LtaS_Lm_-His_6_ (ANG1424) (3), which contains a plasmid for the expression of the C terminally His-tagged LtaS_Lm_ variant, was used for the purification of the secreted eLtaS_Lm_ fragment directly from *Listeria* culture supernatant. This strain was grown overnight in 6 liters of BHI medium. The bacterial cells were pelleted by centrifugation for 10 min at 7,000 × *g* and the cleared culture supernatant was filtered and loaded into a nickel-nitrilotriacetic acid column for protein purification, as previously reported (16). The elution fractions containing the C terminally His-tagged eLtaS_Lm_ protein were pooled together and concentrated to a final volume of ∼50 μl at 0.5 mg/ml using a 10-kDa molecular mass cut-off Centricon. The sample was mixed with an equal volume of protein loading buffer and 5 μg of protein separated on a 12% SDS-PAGE gel alongside 100 μg of eLtaS_Lm_ protein produced and purified from *E. coli* strain ANG1479. Protein bands were visualized by Coomassie staining. The eLtaS_Lm_ protein bands were excised from the gel, digested with chymotrypsin, and subjected to mass spectrometry analysis at the TAPLIN Mass spectrometry facility (Harvard Medical School, Boston, MA). The expected active site threonine containing peptide FHQTGQGKTADSEM (T catalytic threonine) has a calculated mass of 1536.6 Da when unmodified or 1616.6 Da with a phosphorylated threonine residue.

##### Protein Crystallization and Structure Determination

The solubility of eLtaP_Lm_ was 120 mg/ml in 20 mm Tris-HCl, pH 7.5, buffer and most crystallization drops remained clear in the initial screens. To decrease the solubility, the protein was subjected to Lys-methylation (20). Crystals appeared after 7–10 days at 4 °C in 100 mm sodium cacodylate buffer, pH 5.4, 100 mm MgCl_2_, 33% PEG2000 at a protein concentration of 40 mg/ml. Crystals were flash cooled in liquid nitrogen without additional cryoprotection. Non-methylated protein alone failed to produce crystals under these conditions. However, macro-seeding or micro-seeding using the methylated protein promoted crystallization of the non-methylated protein. Therefore a methylated seed stock, stored at stored at 4 °C, was routinely used for seeding. Data were collected at the SOLEIL synchrotron at the PROXIMA1 beamline (Saint-Aubin, France) from a single crystal at 100 K. The crystal belonged to space group P1 with unit cell parameters *a* = 53.20 Å, *b* = 53.70 Å, *c* = 85.07Å; α = 71.57°, β = 87.89°, γ = 65.12°. A mini-κ goniometer was used to obtain high completeness in all resolution shells. Data were indexed with XDS (21) and reduced with SCALA (21, 22) to 1.75-Å resolution. The *R*_free_ set was generated randomly in UNIQUE (23). The structure was solved by molecular replacement using PHASER as implemented in PHENIX AutoMR (24) using, after side chain pruning and ligands removal in SCULPTOR (24), the *S. aureus* eLtaS structure as model (PDB 2W5Q). Initial refinement and model building were performed in PHENIX AutoBuild and completed by cycles of reiterated manual building in COOT (25) and refinement in REFMAC (26). Structure validation was performed using MOLPROBITY (27).

Crystals of eLtaS_Lm_ grew in 5–7 days at 20 °C in 0.64 m sodium acetate, pH 4.6, 4% PEG3350, 100 mm MgCl_2_ and were cryo-protected with 25% PEG400 before flash-cooling in liquid N_2_. A micro-seeding technique was employed to improve the crystal size (28) and crystallization trials were repeated in the same buffer conditions but lowering the protein concentration to 5 mg/ml. For the GroP co-crystallization experiments, the protein was incubated for 10 min at room temperature with a final concentration of 50 mm GroP. The crystals obtained from the co-crystallization were further soaked for 5 min in crystallization buffer supplemented with 25% PEG400 and 50 mm GroP before flash cooling in liquid N_2_. Data collection of the apo-eLtaS_Lm_ was performed at the Diamond Light Source synchrotron, beamline I24 (Didcot, Oxford, UK), from a single crystal at 100 K. The apo-structure of eLtaS_Lm_ belonged to the space group *P*4_1_2_1_2 with unit cell dimensions of *a* = *b* = 119.76 Å, *c* = 473.91 Å; α = β = γ = 90.0°. The data were indexed, scaled, and *R*_free_ was generated randomly in UNIQUE (23). The structure was solved by molecular replacement using BALBES (29) and the *B. subtilis* eLtaS_Bs_ structure (PDB code 2W8D) as a starting model. Rigid body and restrained refinement produced a drop of *R*_factor_ and *R*_free_ from 42 and 43% to 25 and 31%, respectively. The structure was refined and validated as described above for eLtaP_Lm_.

The data collection of the eLtaS_Lm_-GroP complex was performed at the Diamond Light Source synchrotron beamline I04-1 (Didcot, Oxford, UK) from a single crystal at 100 K. The crystals belonged to the space group *P*2_1_2_1_2_1_ with unit cell dimensions of *a* = 119.25 Å, *b* = 119.63 Å, *c* = 472.66 Å; α = β = γ = 90.0°. Indexing was performed in XDS and data merging was performed in SCALA and TRUNCATE (23) H- and L-test analysis in TRUNCATE highlighted the presence of pseudo-meroheydral twinning. The *R*_free_ set was generated randomly in UNIQUE and the structure was solved by molecular replacement in PHASER using apo-eLtaS_Lm_ as a model. Ten cycles of rigid body refinement (10.0–6.0 Å) followed by 10 cycles of restrained refinement in REFMAC gave an *R* value of 23.6% and *R*_free_ of 25.0%. Twin refinement in REFMAC highlighted a twin fraction of 9% with twinning operator *k*, *h*, -*l*. Therefore the twin option was kept for the whole refinement process, which was iterated with manual building in COOT. The final step of the refinement with rotamer optimization was performed in PHENIX, which did not detect any twinning. Composite omit maps were calculated in PHENIX and used to orient the terminal OH group of GroP. Structure validation was performed using MOLPROBITY. Ligand coordinate and dictionary files were generated and regularized in JLIGAND (30). Anomalous maps were generated using the SFTOOLS (23) and visualized in PYMOL. The statistics for all data sets are shown in Table 3.

**TABLE 3:** **Dataset statistics** The information for the last shell of resolution is given in parentheses.

	eLtaP apo	eLtaS apo	eLtaS-GroP
Synchrotron	Soleil	Diamond	Diamond
Beamline	Proxima1	I24	I04–1
Space group	*P*_1_	*P*4_1_2_1_2	*P*2_1_2_1_2_1_
*a*, *b*, *c* (Å)	53.20	119.76	119.25
α, β, γ (^o^)	53.70	119.76	119.63
	85.04	473.91	472.66
	71.63	90.00	90.00
	76.78	90.00	90.00
	65.12	90.00	90.00
Resolution (Å)	47.96 (1.84-1.75)	106.88 (3.16-3.00)	48.71 (2.32-2.20)
*R*_merge_*^a^*	0.069 (0.501)	0.117 (0.560)	0.089 (0.459)
*R*_pim_*^b^* (all I^+^ and I)	0.040 (0.293)	0.062 (0.318)	0.052 (0.265)
〈*I*〉/SD 〈*I*〉	10.7 (2.5)	8.6 (2.3)	11.5 (2.7)
Completeness (%)	94.8 (92.6)	90.7 (84.6)	98.2 (95.7)
Redundancy	3.8 (3.8)	4.0 (3.5)	3.8 (3.8)
No. reflections	293,738 (41,866)	257,649 (29941)	1,285,177 (180393)
No. unique	77,013 (11,049)	63,877 (8,525)	335,456 (47,358)
*R*_factor_*^c^*	0.178 (0.296)	0.222 (0.319)	0.178 (0.208)
*R*_free_*^d^*	0.207 (0.330)	0.260 (0.376)	0.214 (0.237)
No. atoms	7,119	16,745	40,238
Protein	6,604	16,740	37,131
Water	481		2,997
Ligands	34	5	110
Average B-factors (Å^2^)	31.4	50.9	33.1
Protein	30.9	50.9	33.0 (30.6)*^e^*
Waters	37.0		33.7
Ligands	44.7	45.5	38.6
Root mean square deviations			
Bond lengths (Å)	0.009	0.006	0.007
Bond angles (°)	1.23	0.93	1.07
Ramachandran most favored (%)	97	97	98
Ramachandran additional allowed (%)	3	3	2
Ramachandran outliers (%)	0	0	0
PDB code	4UOP	4UOO	4UOR

*^a^ R*_merge_ = Σ*_h_*Σ*_l_* |*I_hl_* − 〈*I_h_*〉|/Σ*_h_*Σ*_l_* 〈*I_h_*〉, where *I_hl_* is the *I*th observation of reflection h and 〈*I_h_*〉.

*^b^ R*_pim_ as described in Ref. 43.

*^c^ R*_factor_ = Σ*_h_*‖*F*_obs(_*_h_*_)_| − |*F*_cal(_*_h_*_)_‖/Σ*_h_*|*F*_obs(_*_h_*_)_, where *F*_obs(_*_H_*_)_ − *F*_cal(_*_h_*_)_ are the observed and calculated structure factors for reflection *h*, respectively.

*^d^ R*_free_ factor was calculated same as *R*_factor_ using 5% reflections, which were selected randomly and omitted from refinement.

*^e^* B-factor calculated excluding the disordered monomer K.

##### One-dimensional ^1^H NMR Analysis of eLtaP_Lm_

10 mg of eLtaP_Lm_ in 1 ml of 20 mm Tris-HCl, pH 7.5 buffer, was used for the one-dimensional ^1^H NMR analysis. 10% D_2_O was added to the protein sample and the spectra were recorded at 800 MHz at 37 °C before and after the addition of 10 mm EDTA final concentration.

##### Modeling of the GroP Trimer in the Catalytic Site of eLtaS_Lm_

The coordinate and restraint files of the GroP trimer in its energy minimized form were generated with JLIGAND (30). Superposition of the coordinates of the GroP trimer with the eLtaS-GroP complexes was performed in PYMOL.

##### Enzyme Activity Assay

The activity of wild-type eLtaS_Lm_ and eLtaS_Lm_ variants T307A, S486A, N488A, H489A, and S486A/N488A/H489A was determined as previously reported (11). Briefly, 4 μg of the fluorescently labeled NBD-PG lipid substrate was incubated for 3 h at 37 °C with 30 μg of enzyme in 10 mm sodium succinate buffer, pH 6.0, adjusted to an ionic strength of 50 with NaCl and 10 mm MnCl_2_. The lipid reaction products were subsequently extracted with chloroform and methanol, separated by thin layer chromatography, and the signal of the NBD-DAG hydrolysis product quantified as previously described (11). Each TLC plate contained a negative no-enzyme control lane to determine the background signal, as well as a wild-type eLtaS_Lm_ enzyme reaction, which was for normalization purposes set to 100%. The activity of the different variants was calculated as percentage of activity compared with the wild-type control reaction. Four independent experiments with two different protein purifications were performed and the average percentage of activity and standard deviation were plotted.

##### LTA and Protein Detection by Western Blot

The different *L. monocytogenes* strains were grown overnight at 30 °C in BHI medium. Sample analysis for the detection of LTA or the His-tagged LtaS variants by Western blot was performed as previously described (3).

##### Listeria Growth Curves and Microscopy Analysis

The indicated *L.* monocytogenes strains were grown overnight at 30 °C in BHI medium. The next day, the cultures were back diluted to an *A*_600_ of 0.05, incubated at 37 °C with shaking, and growth was monitored by determining *A*_600_ readings at timed intervals. For microscopy analysis, the different *L. monocytogenes* strain was propagated for at least 6 h at 37 °C in BHI medium. Subsequently culture aliquots were adjusted to an *A*_600_ of 0.5 and analyzed by phase-contrast microscopy using a Nikon Eclipse TS100 microscope with a ×20 objective. Images were recorded using a Sony HDR-CX11 high-definition camcorder mounted onto the microscope. Two independent microscopy experiments and three independent growth curves were performed and representative results are shown.

##### Bioinformatics and Sequence Analysis

Sequences homologous to the full-length LtaS and LtaP sequences were retrieved from the RefSeq microbial non-redundant database (31) using PSI-BLAST (32) with an *E*-value cutoff 1e-40. Sequences were filtered to have an alignment length of at least 400 residues, an identity of at least 28.7%, and similarity of 48.5% to either LtaS or LtaP. These cutoff values were chosen as they are the sequence identity and similarity between LtaS and LtaP. Sequences with a higher similarity to LtaP than LtaS were assigned to a primase-like sequence list (50 sequences), whereas sequences with a higher similarity to LtaS were assigned to a synthase-like list (1038 sequences). The LtaP and LtaS sequences were separately aligned using MUSCLE (33) and then combined using MUSCLE profile-profile alignment. The phylogenetic tree using the combined alignment (having removed any columns not aligned to either LtaS or LtaP) was generated using the program PROML from PHYLIP version 2.3 (34) and plotted using the R package APE (35). All logo plots were produced using WebLogo (36). For the PSICOV (37) amino acid covariation analysis a new larger alignment was produced of LtaS_Lm_ homologous retrieved from the non-redundant database using PSI-BLAST and an *E*-value cutoff of 10^−10^. These sequences were individually aligned to the LtasS_Lm_ sequence using the BLOSUM62 matrix and Smith-Waterman algorithm, insertions were removed and pairwise alignments were combined to produce a multiple sequence alignment. Redundant sequences and sequences covering less than 60% of the LtaS_Lm_ sequence were removed resulting in 6943 final sequences. This final alignment was subsequently analyzed using the residue contact prediction program PSICOV (37).

## RESULTS

### 

#### 

##### Apo-structures of eLtaP_Lm_ and LtaS_Lm_

**FIGURE 1.**
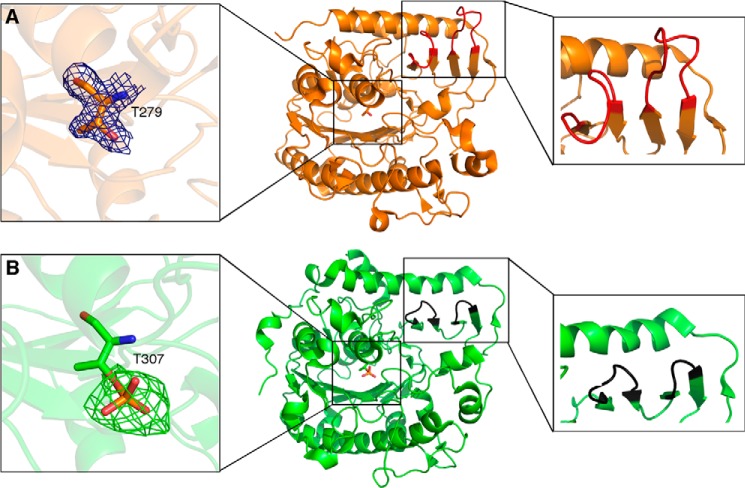
**Crystal structures of apo-eLtaP_Lm_ and apo-eLtaS_Lm_.** Schematic representation of the crystal structures of eLtaP_Lm_ (*A*) and eLtaS_Lm_ (*B*) with close ups of the catalytic Thr residues (*left panel*) and loop regions (*right panel*). The structural differences in the loop regions between eLtaP_Lm_ and eLtaS_Lm_ are highlighted in *red* and *black*, respectively. The 2*F_o_* − *F_c_* electron density map (1.0 root mean square deviation) of Thr-279 in eLtaP_Lm_ is highlighted in *blue* and the omit F*_o_* − *F_c_* map (3.0 root mean square deviation) of the phospho-Thr-307 in eLtaS_Lm_ is highlighted in *green*.

**FIGURE 2.**
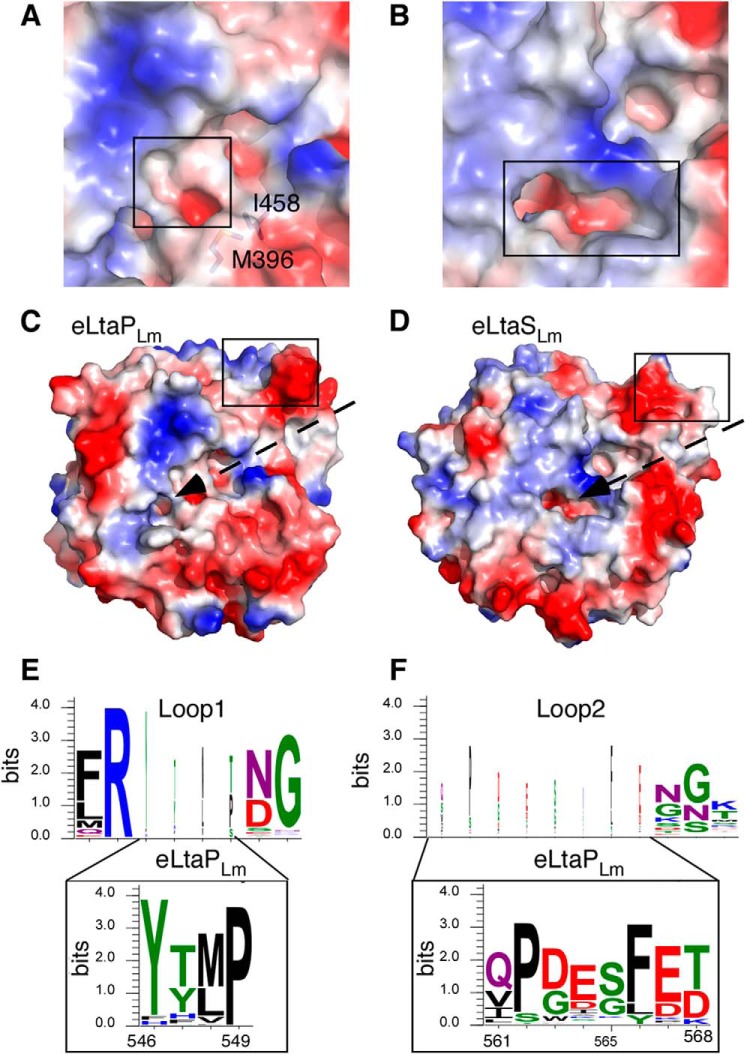
*A* and *B,* comparison of the catalytic pocket of eLtaP_Lm_ and eLtaS_Lm_. Surface potential representation (*blue*, positive; *red*, negative; *white*, hydrophobic) of the area around the catalytic site of eLtaP_Lm_ (*A*) and eLtaS_Lm_ (*B*). The catalytic pocket of eLtaP_Lm_ is restricted through the highlighted amino acids Met-396 and Ile-458 and therefore significantly smaller and more hydrophobic than in eLtaS_Lm_. *C* and *D,* surface potential representation of eLtaP_Lm_ (*C*) and eLtaS_Lm_ (*D*) structures with insertion loop regions *boxed* and a hydrophobic groove (*white*) stretching from loop 2 to the active site in eLtaP_Lm_ as indicated. *E* and *F*, web logo motif for the insertion loop 1 (*E*) and insertion loop 2 (*F*) region of the top 1090 LtaS-type sequences shown on *top* and the web logo motif for the 51 LtaP-type sequences shown *below*, using amino acid numbering for LtaP_Lm_. The dimension of the letters in WebLogos are directly proportional to the degree of conservation of the given residue.

To identify differences between LTA synthase and primase enzymes, the soluble extracellular enzymatic domains eLtaP_Lm_ and eLtaS_Lm_ were overexpressed and purified from *E. coli* and their crystal structures were determined at 1.75- and 3.0-Å resolution, respectively. Although both enzymes were monomers in solution, as assessed by size exclusion chromatography, eLtaP_Lm_ crystallized with two molecules in the asymmetric unit and eLtaS_Lm_ with five molecules in the asymmetric unit (Table 3). The overall structures of eLtaS_Lm_ and eLtaP_Lm_ are very similar (root mean square deviation = 1.4 Å for Cα atoms). Both comprise an α/β core and a C-terminal part of four anti-parallel β-strands and a long α-helix (Fig. 1). As predicted, both enzymes are similar to eLtaS_Sa_ (PDB code 2W5Q) and eLtaS_Bs_ (PDB code 2W8D) with a root mean square deviation on Cα atoms of 1.7 Å for eLtaP_Lm_ and 0.9 Å for eLtaS_Lm_. Although the electrostatic surface potentials of eLtaS_Lm_ and eLtaP_Lm_ are similar around the α/β core at the N-terminal end, there are substantial differences in cavity size and surface charge distribution around the catalytic centers (Fig. 2, *A* and *B*).

A structure/sequence comparison of the two enzymes highlighted two sequence insertions in LtaP that form two extended loops (residues 544–552, loop 1; residues 561–570, loop 2), which interact with the long helix α18 (Figs. 1 and 2). There is no sequence conservation in loop 1 and loop 2 between eLtaP_Lm_ and eLtaS_Lm_ except for the salt bridge formed by residues Asp-600 and Arg-545, which correspond to Asp-616 and Arg-576 in the synthase enzyme. The insertion loop 2 in eLtaP_Lm_ forms a negatively charged protrusion, which is repositioned through Phe-566 on α18 by ∼2 Å compared with eLtaS_Lm_. This also leads to the formation of a surface groove, which extends to the catalytic site (Fig. 2*C*). In eLtaS_Lm_, this surface groove is constricted by Lys-306, which form a hydrogen bond with Tyr-483 (Fig. 2*D*). The specific loop 1 and loop 2 sequence insertions are conserved within primase homologues (Fig. 2, *E* and *F*) suggesting that the resulting surface features are specific for the function of primase enzymes.

##### The Catalytic Threonine Is Unmodified in the Natural Host

**FIGURE 3.**
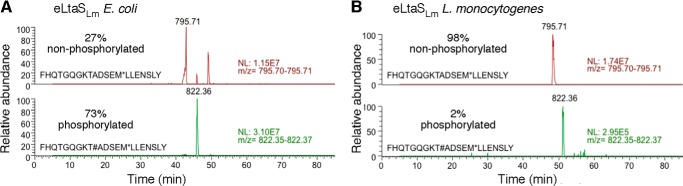
**Phosphorylation state of active site Thr as determined by mass spectrometry.** *A,* eLtaS_Lm_ was purified from the *E. coli* cytoplasm or *B,* directly from the supernatant of a *L. monocytogenes* culture, separated on an SDS-PAGE gel, and subjected to a chymotrypsin digest and mass spectrometry analysis. The mass spectrometry traces corresponding to the active site containing peptide are shown for eLtaS_Lm_ purified from *E. coli* (*A*) or *L. monocytogenes* (*B*). The expected active site threonine containing peptide FHQTGQGKTADSEM (T, catalytic threonine) has a calculated mass of 1536.6 Da when unmodified or 1616.6 Da with a phosphorylated Thr residue. The fraction of protein with a phosphorylated active site Thr was estimated based on the intensity of the mass spectrometry signal and is indicated in % in each panel.

The catalytic residue of LtaS-type enzymes is a highly conserved Thr residue that in the *B. subtilis* eLtaS_Bs_ structure is phosphorylated but unmodified in the *S. aureus* eLtaS_Sa_ structure (13, 18). In this study, we found that Thr-307 in eLtaS_Lm_ is phosphorylated, whereas the corresponding residue Thr-279 in eLtaP_Lm_ is unmodified (Fig. 1). To gain insight into the physiological relevance of this modification, a C terminally His-tagged LtaS_Lm_ variant was expressed in *L. monocytogenes* and the cleaved eLtaS_Lm_ domain was purified from the culture supernatant. The purified protein was digested with chymotrypsin, and peptide fragments were analyzed by electron spray mass spectrometry. This analysis showed that for eLtaS_Lm_ expressed in *E. coli* the catalytic Thr is mostly phosphorylated (73%), whereas only 2% of the protein purified from the natural host is phosphorylated (Fig. 3). These data suggest that phosphorylation of the catalytic Thr is not physiological but is likely a result of expression in a heterologous host. However, as shown below this modification is likely a mimic of an enzyme-substrate intermediate.

##### Preferential Binding of Mn^2+^ to the Conserved Metal Binding Site

**FIGURE 4.**
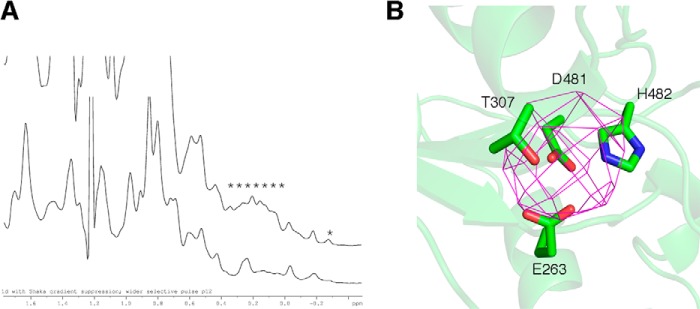
**Characteristics of enzyme-bound metal.** *A*, one-dimensional ^1^H NMR spectra of eLtaP_Lm_ in the absence or presence of EDTA one-dimensional NMR spectra of purified eLtaP_Lm_ protein recorded on a 800 MHz magnet at 37 °C before and after addition of 10 mm EDTA. *B,* anomalous electron density map of eLtaS_Lm_. eLtaS_Lm_ crystals were grown in the absence of any added metal ions and data collected close to the Mn^2+^ edge (1.28 Å). The DANO SigDANO electron density map (shown in *purple*) confirms the presence of a Mn^2+^ ion.

LtaS-type proteins are metal-dependent enzymes and the highest *in vitro* enzyme activity is observed in the presence of Mn^2+^ (11, 17). Our data show that the metal binding site is identical in the LtaS_Lm_ and LtaP_Lm_ structures. In previous LtaS crystal structures both Mn^2+^ and Mg^2+^ were identified in the metal binding site near the catalytic threonine, facilitating phosphatidylglycerol hydrolysis (13, 18). As the crystallization buffer for both *Listeria* proteins contained a high MgCl_2_ concentration, it is likely that Mg^2+^ is present in the active center in our structures. To determine the metal preference of the enzymes, crystallization trials were set up in the absence of any added metal ion. Although the eLtaP_Lm_ protein did not crystallize under these conditions, one-dimensional ^1^H NMR experiments showed an increase in peak sharpness upon addition of EDTA, suggesting the presence of a paramagnetic ion such as Mn^2+^ (Fig. 4*A*). Although the eLtaS_Lm_ crystals grown in the absence of any added metal ion diffracted only to 6.4 Å, anomalous difference maps showed a strong anomalous peak consistent with the presence of a bound Mn^2+^ ion after expression and purification (Fig. 4*B*). Together our data provide evidence for preferential Mn^2+^ binding of both eLtaP_Lm_ and eLtaS_Lm_, in the absence of added metals consistent with previous biochemical activity measurements.

##### Identification of GroP Binding Sites in eLtaS_Lm_

LtaS-type enzymes belong to the arylsulfatase group of enzymes and the reaction mechanism of other members of this class of enzymes proceeds through the formation of a covalent enzyme-substrate intermediate. In the case of sulfatases, a post-translationally modified cysteine residue, a hydroxyformylglycine, is sulfated during catalysis (38). We previously speculated that LtaS-type enzymes also form a covalent GroP-Thr intermediate as part of the reaction mechanism (13). Although we show here that the phosphorylation of the active site Thr residue observed in the eLtaS_Lm_ structure does not occur in the native host (Fig. 3), its presence in *E. coli* could, however, mimic such a covalent enzyme substrate intermediate. To provide additional experimental evidence for the formation of a covalent GroP-Thr intermediate, we performed co-crystallization and crystal soaking experiments with the eLtaS_Lm_ and PG lipid substrates with short chain fatty acids. However, co-crystallization experiments failed to produce crystals and crystal-soaking experiments abolished the diffraction power of the crystals. Next, co-crystallization and soaking experiments were performed with GroP, the hydrolysis product of the lipid substrate PG, and the structure was solved from crystals containing 11 molecules in the asymmetric unit.

**FIGURE 5.**
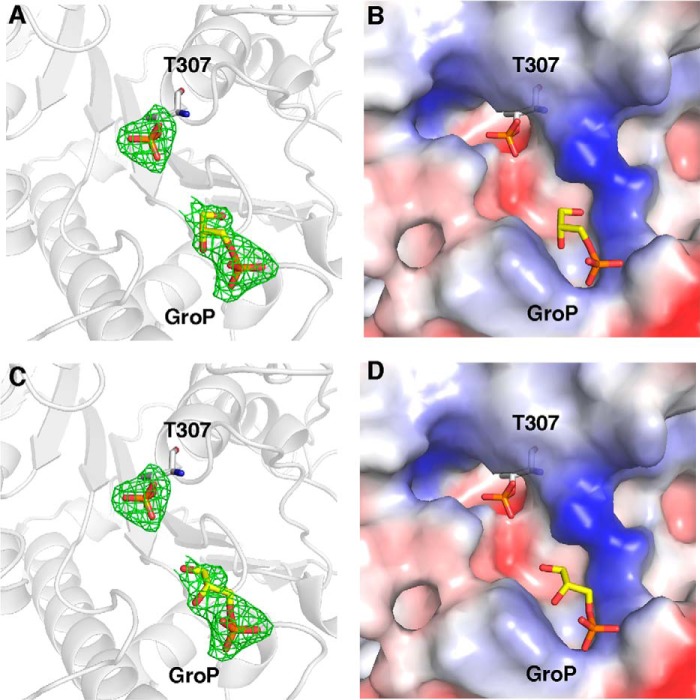
**GroP/eLtaS_Lm_ co-crystal structure.** *A,* ribbon representation of the active site of eLtaS_Lm_ with bound phosphate and a GroP molecule in a second site, as observed in chains B, C, D, F, H, I, J, and K. The phosphate group is covalently linked to the catalytic Thr-307 and GroP is bound at the entrance of the catalytic site. The omit *F_o_* − *F_c_* electron density map (3.0 root mean square deviation) of the phospho-threonine (*T307*) and GroP are shown in *green. B*, electrostatic surface potential representation (*blue*, positive; *red*, negative) around the catalytic site showing the charge distribution surrounding the phospho-Thr-307 and the GroP molecule. *C,* ribbon representation of the active site of eLtaS_Lm_ with bound phosphate and a GroP molecule in a second site (as observed in chains A, E, and G) and *D,* the corresponding surface potential representation.

**FIGURE 6.**
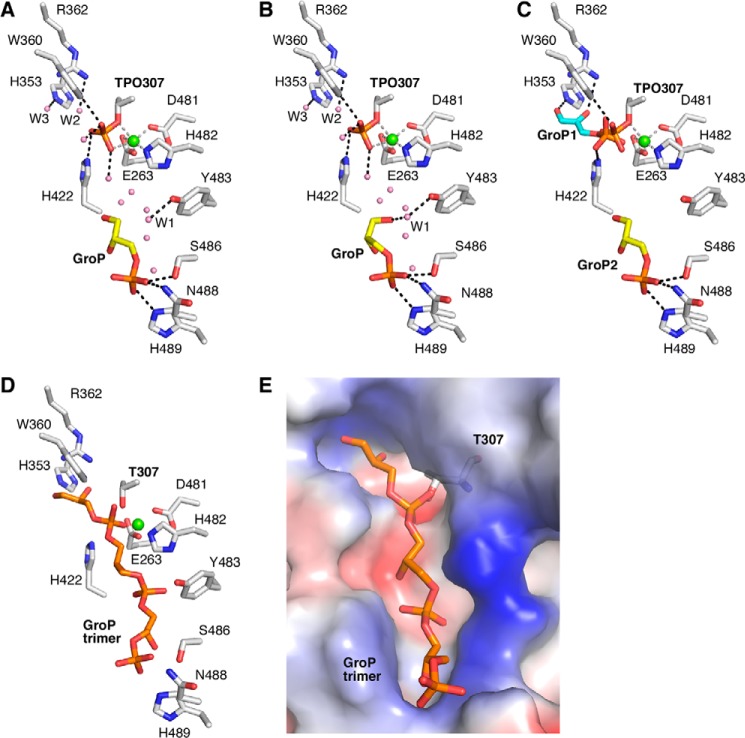
**Detailed view of the active center with metal and ligand interactions.** *A,* structural details of the eLtaS_Lm_ active site showing the phospho-Thr intermediate (*TPO307*) and the metal binding site (as seen in chains A, E, and G). Hydrogen bonds are indicated as *gray lines* for the metal binding site and as *black lines* for the ligands. The water molecules that are conserved in all 11 monomers are represented as *spheres* in *pink*. Residues His-353, Arg-362, Trp-360, His-422, and two water molecules are involved in the binding of the phosphate group and phospho-Thr-307, Glu-263, Asp-481, and His-482 coordinate the Mg^2+^ ion. The GroP molecule, which is 6.5 Å removed from the catalytic residue, forms hydrogen bonds with Ser-486, Asn-488, and His-489. *B,* structural details of the eLtaS_Lm_ active site showing the phospho-Thr intermediate (*TPO307*) and the metal binding site (as observed in chains B, C, D, F, H, I, J, and K). The GroP molecule forms hydrogen bonds with Ser-486, Asn-488, and His-489 like in *panel A* but in addition it also binds to a water molecule (*W1*), which in turn forms a hydrogen bond with Tyr-483. *C,* superposition between the catalytic site of eLtaS_Lm_ and the GroP molecules trapped in the eLtaS_Sa_-T300A structure (PDB code 2W5R). *D,* model of the eLtaS_Lm_ active site bound to a GroP trimer. The GroP timer was produced and minimized with JLIGAND and superposed in PYMOL on the experimental crystal structure of the GroP-eLtaS_Lm_ co-crystal structure. *E,* electrostatic potential representation of the eLtaSLm active site with the modeled GroP trimer. For clarity, the image is rotated by +30° around the *y* axis compared with *panel D*.

Using this approach, extra electron density was observed in each monomer within the catalytic site (Fig. 5). Similar as in the apo-structure, it was possible to build a phosphate group into a density extending from Thr-307 (Fig. 5). The phosphate oxygen binds to two structurally conserved water molecules, Trp-360, His-422, and a Mg^2+^ ion that is in turn further coordinated by Glu-263, Asp-481, and His-482 (Fig. 6, *A* and *B*). Additional difference electron density was observed in each monomer at the entrance of the catalytic pocket, into which a GroP molecule could be built (Fig. 5). In all chains, the phosphate group of the GroP molecule in this second site formed hydrogen bonds with residues Ser-486, Asn-488, and His-489 (Fig. 6, *A* and *B*). In eight molecules in the asymmetric unit an additional hydrogen bond was observed between the terminal hydroxyl group of GroP and a water molecule (W1), which in turn forms a hydrogen bond with Tyr-483 (Fig. 6*B*). In a previous study, the co-crystal structure of the *S. aureus* active site variant eLtaS_Sa_-T300A with a GroP molecule within the active center was determined (PDB code 2W5R) (13). The overlay of the catalytic sites of the GroP-eLtaS_Sa_-T300A and the GroP-eLtaS_Lm_ structures revealed that the GroP molecule within the active center (referred to as GroP1) superposed with the phosphothreonine and the conserved water molecules W2 and W3 in eLtaS_Lm_ (Fig. 6*C*). Therefore, the phosphorylated Thr likely mimics a covalent GroP-Thr intermediate. The distance between the phosphorylated Thr and the terminal hydroxyl group of the GroP2 molecule bound at the entrance of the catalytic pocket is ∼6.3 Å, which is compatible with the length of one intervening GroP molecule. To test whether an additional GroP molecule could fit into this space, a GroP trimer model was generated *in silico* and fitted into the eLtaS_Lm_ structure using the experimental electron densities of the phosphothreonine and GroP as a guide (Fig. 6*D*). Our modeling showed that a GroP could fit in the intervening space suggesting that the growing PGP LTA chain could be bound in a similar manner during the catalytic cycle of eLtaS_Lm_. The nature of the surface potential of the oligo-GroP binding groove further supports this conclusion (Fig. 6*E*). A series of ordered water molecules spans the catalytic site of eLtaS_Lm_ from residue His-353 to the trapped GroP2 molecule. The positions of these water molecules are conserved across all 11 monomers within a crystallographic unit and trace the position of the modeled GroP trimer (Fig. 6).

##### The Second GroP Binding Site in eLtaS_Lm_ Is Essential for Enzyme Function

**FIGURE 7.**
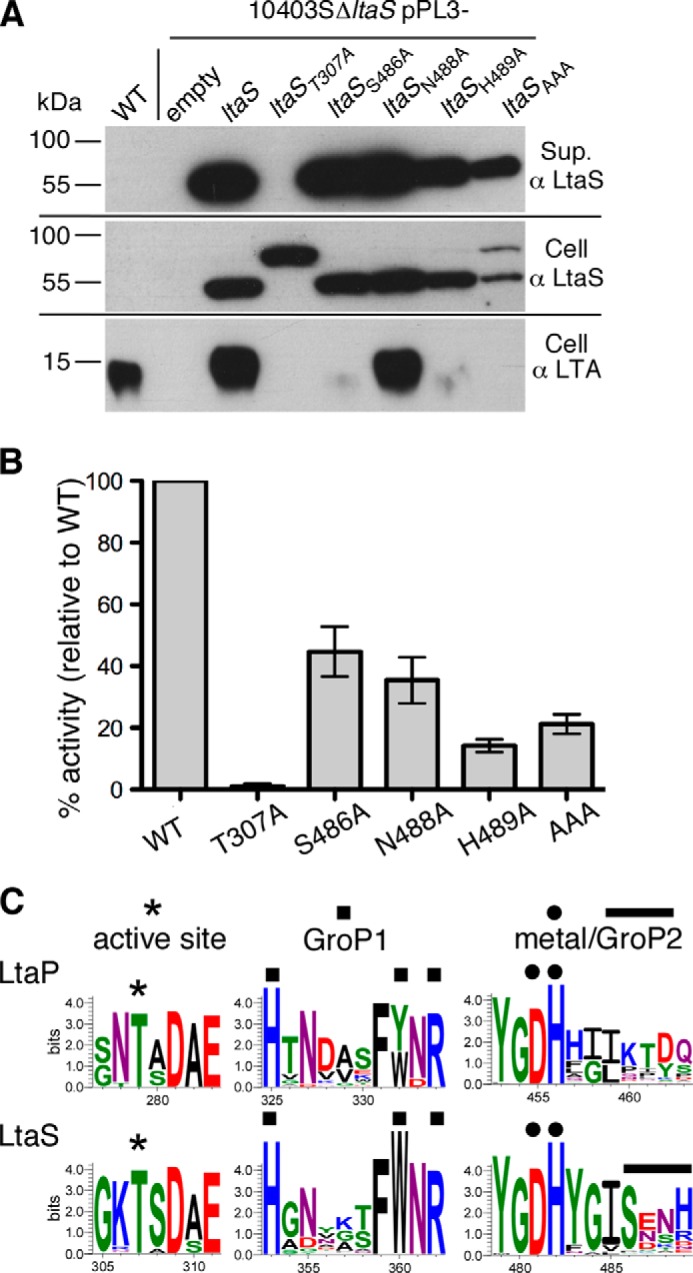
***In vivo* and *in vitro* activity of eLtaS_Lm_ GroP binding site variants and bioinformatics analysis of conserved binding residues.** *A*, detection of LtaS_Lm_ protein and LTA by Western blot. Samples of wild-type *L. monocytogenes* 10403S (*WT*) and the 10403SΔ*ltaS*-derived strains containing an empty pPL3 vector or expressing the indicated LtaS_Lm_ variants as C-terminal His tag fusion proteins were prepared for Western blot analysis. The LtaS protein was detected in the supernatant and cell wall-associated fractions using a His tag-specific antibody and LTA in the cell wall-associated fraction using a polyglycerolphosphate-specific antibody. *B*, *in vitro* enzyme activity assay with purified WT eLtaS_Lm_ and the different eLtaS variants. Enzyme reactions were set up using the fluorescently labeled lipid NBD-PG as substrate. The reaction products were separated on TLC plates and the NBD-DAG product was quantified. Four independent experiments were performed and the enzyme activity of the eLtaS_Lm_ protein (labeled WT in the graph) was set to 100% in each experiment. The relative activity of the different variants compared with WT eLtaS_Lm_ was calculated and the average value and S.D. plotted. *C*, sequence logo motif of active site, metal binding, active site GroP (GroP1) and second GroP (GroP2) binding site residues. The 51 LtaP-like sequences (*top panels*) and the 1039 LtaS-type sequences (*bottom panels*) were aligned and logo motifs for selected amino acid regions are shown. Active site residue (*), GroP1 (■), GroP2 (−), and metal binding residues (●) are indicated and amino acid numbering for the respective *L. monocytogenes* protein is shown.

To test the functional requirement of the second GroP binding site, we mutated residues Ser-486, Asn-488, and His-489 to alanines individually or in combination and tested the mutant enzymes for their ability to produce LTA (Fig. 7). The different variants were expressed as C-terminal His tag fusion proteins in the *L. monocytogenes* strain 10403SΔ*ltaS*, which contains a deletion of the native *ltaS* gene. As negative controls, an empty vector or a vector for the expression of the catalytic site variant T307A (pPL3-*ltaS*_T307A-His6_) were introduced into 10403SΔ*ltaS* and as positive control a vector for expression of wild-type LtaS (pPL3-*ltaS*_His6_). Expression of all LtaS variants was confirmed by Western blot. As previously reported for WT LtaS_Lm_ (3), all GroP binding site variants were cleaved and the eLtaS fragment was detected in the culture supernatant as well as in the cell wall-associated fraction (Fig. 7*A*). The active site T307A variant remained unprocessed and the full-length protein was observed in the cell wall-associated fraction (Fig. 7*A*). In a previous study, a similar accumulation of the full-length protein was observed in *S. aureus* for the catalytic site variant (13), suggesting that an enzyme/substrate intermediate is required to position the enzyme for efficient processing. However, it should also be noted that the protein processing step does not serve as an enzyme activation step; to the contrary, based on experiments performed in *S. aureus* it has been proposed that the LtaS cleavage step serves as a mechanism to inactivate the enzyme (16).

As expected, LTA production was restored to wild-type levels in the positive control strain 10403SΔ*ltaS* pPL3-*ltaS*_His6_, whereas no LTA-specific signal was detected when extracts from the negative control strains were analyzed by Western blot using a polyglycerolphosphate-specific monoclonal LTA antibody (Fig. 7*A*). Expression of the S486A/N488A/H489A variant (LtaS_AAA_) did not restore LTA production, revealing an essential function of the second GroP binding site for LTA production. Analysis of the single amino acid variants showed that residues Ser-486 and His-489, but not Asn-488 are important for the LTA polymerization step (Fig. 7*A*).

For successful LTA production, PG substrate hydrolysis and the GroP transfer reaction must take place. To determine whether the second GroP binding site is required specifically for PG hydrolysis, the WT and different eLtaS variants were produced in *E. coli*, purified, and used for *in vitro* enzyme reactions with fluorescently labeled NBD-PG lipid as substrate. As expected, mutating the catalytic Thr-307 residue abolished enzyme activity (Fig. 7*B*). The S486A and N488A variants retained the ability to hydrolyze PG, but the activity dropped by ∼50% compared with wild-type eLtaS_Lm_. The H489A and S486A/N488A/H489A (AAA) variants showed a marked decrease in activity to around 20% of WT (Fig. 7*B*). These data show that the second GroP binding site, in particular residue His-489, is also important for the PG hydrolysis step. The S486A variant, however, is of particular interest as this variant retains significant PG hydrolysis activity, whereas the PGP polymerase activity is nearly abolished. We would suggest that this is due to the inability of this variant to interact with the growing PGP chain and therefore, similar to what is observed naturally in the LTA primase enzyme, the two reactions are decoupled in this variant.

**FIGURE 8.**
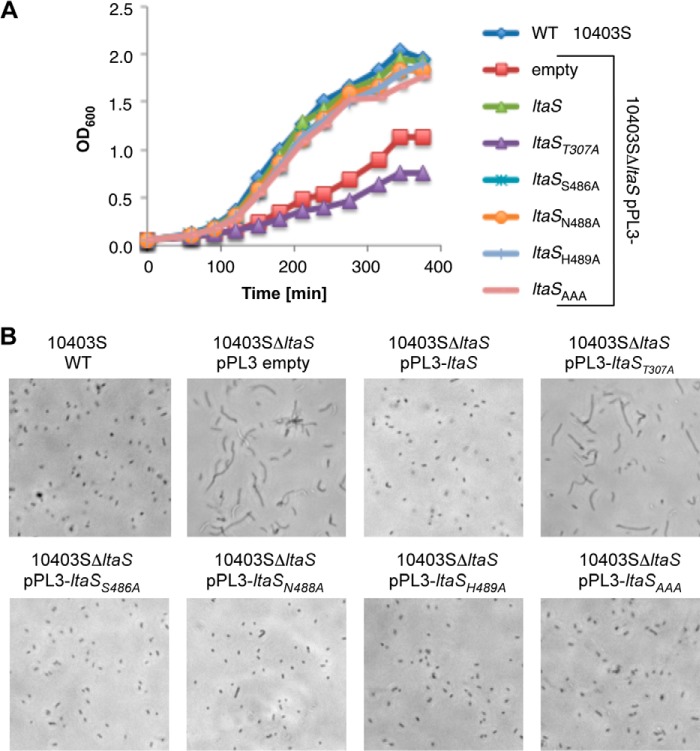
**Growth and microscopy analysis of wild-type *L. monocytogenes*, mutant, and complementation strains.** *A,* growth curves. The wild-type *L. monocytogenes* strain 10403S (WT) and 10403SΔ*ltaS*-derived strains containing an empty pPL3 vector or a pPL3 vector with the indicated *ltaS* allele were grown at 37 °C in BHI medium, *A*_600_ readings determined at timed intervals and plotted. *B*, microscopy analysis. The same strains as used for growth curves in *panel A* were analyzed by phase-contrast microscopy following growth at 37 °C.

In a previous study, it has been shown that strain 10403SΔ*ltaS* has growth and morphological defects when propagated at 37 °C (3). To investigate if expression of any of the LtaS variants allows for sufficient LTA production to restore these defects, growth and microscopy analysis was performed with the complementation strains. As expected the *ltaS* deletion strain displayed the expected growth defect and a filamentation phenotype, which could be complemented by introducing a wild-type *ltaS* allele (Fig. 8). For the other complementation strains, only expression of the *ltaS*_T307A_ allele did not restore the growth (Fig. 8*A*) and morphological defects (Fig. 8*B*). These results suggest that even if no signal for LTA is detected by Western blot, limited LTA synthesis must take place in these strains, which is sufficient to support normal growth and cell division.

##### Bioinformatics Analysis and Structure Guided Identification of LtaP and LtaS Enzyme Family Motifs

**FIGURE 9.**
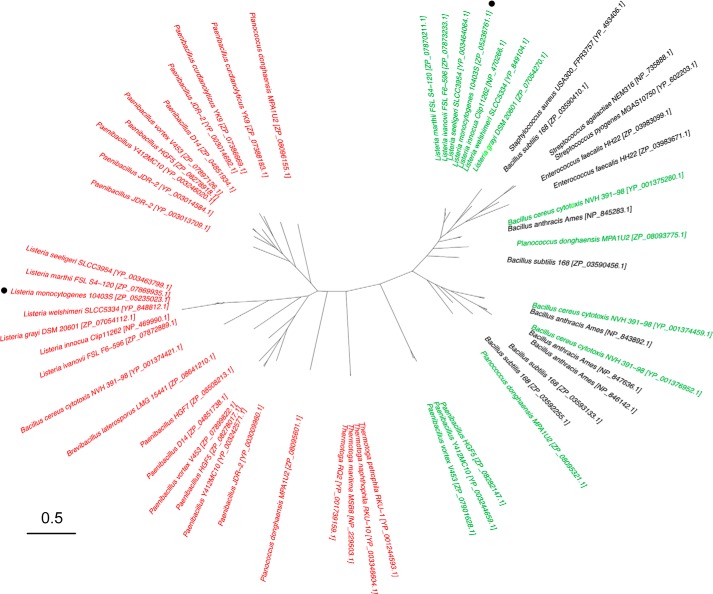
**Unrooted phylogenetic tree of representative LtaS and LtaP-type enzymes.** 1088 LtaS and LtaP sequence homologues were retrieved as described under “Experimental Procedures.” An unrooted phylogenetic tree was generated for representative LtaS and LtaP-type enzymes. Thirty of 50 LtaP-like protein sequences are shown in *red* and 28 of the remaining 1038 LtaS-like sequences are shown in *green* if the same bacterial stain also contains an LtaP-like enzyme or in *black* if the bacterial strain only contains LtaS-like enzymes. For clarity, the majority of the LtaS-type sequences, which would fall onto the right side of the tree, are not shown. The *L. monocytogenes* 10403S proteins analyzed in this study are indicated with *dots*. The *scale bar* indicates the branch length unit of the tree as inferred using the program PROML and is the expected fraction of amino acids changed. A complete list of the organisms and RefSeq accession numbers can be found in supplemental Table S1 using the same color-coding with primase-like sequences shown in *red* and synthase-like sequences shown in *green* or *black*.

To obtain an overview of the distribution of LtaP and LtaS-type enzymes among Gram-positive bacteria and to investigate the conservation of the structural features identified in this study, bioinformatics analyses were performed. To this end, homologues to full-length LtaS and LtaP sequences were retrieved and filtered to those with an alignment length of more than 400 residues yielding 1088 sequences. This was done to remove proteins that do not contain an N-terminal membrane domain and are therefore unlikely involved in LTA production. Of the 1088 retrieved sequences, only 50 showed greater homology to LtaP than to LtaS (supplemental Table S1). Primase family enzymes are present in the different *Listeria* species and similar to *L. monocytogenes* these species also contain an LtaS-type enzyme. This analysis highlighted that a two-enzyme LTA synthesis system with highly divergent enzymes as seen in *Listeria* sp. is not widely distributed among bacteria (Fig. 9 and supplemental Table S1). For instance, *Bacillus* sp. also contain multiple enzymes, but they are more closely related to one another than to the two enzymes found in *Listeria* sp (Fig. 9 and supplemental Table S1). This could indicate that either a gene duplication event took place more recently in *Bacillus* sp. or that the divergent primase-like enzyme was only retained in a few species such as *Listeria, Thermotoga*, and *Paenibacillus* sp. Primase-like enzymes also appear to be present in a few specific bacterial strains such as *Planococcus donghaensis* MPA1U2, *Brevibacillus laterosporus* LMG, and *B. cereus cytotoxis* NVH 391–98 (Fig. 9 and supplemental Table S1). The latter strain was isolated from a fatal case of enteritis. It is therefore plausible that the gene coding for the primase enzyme was acquired through horizontal gene transfer from a *Listeria* strain by co-inhabiting the same ecological niche. It is also of note that the *Thermotoga* sp., *B. laterosporus* LMG, and several of the *Paenibacillus* sp. do not contain an LtaS-type enzyme and hence are unlikely to produce an actual LTA polymer.

As shown above, we have identified a second GroP binding site in LtaS_Lm_ and confirmed its importance for LTA production experimentally. Next, we analyzed distribution of binding site residues Ser-486, Asn-488, and His-489 across LTA synthesis enzymes. Separate alignments were produced for the 1038 LtaS-type sequences and the 50 LtaP-type sequences. Subsequently, a logo motif was created to visualize the conservation of amino acids across the whole enzyme family (data not shown). As expected, the active site threonine, as well as the metal binding residues, were highly conserved and present in both LtaP and LtaS-type enzymes (Fig. 7*C*). In addition, conserved residues in the active site, which are required for binding of the GroP molecule within the active center, could also be identified in both enzyme types (Fig. 7*C*). The second GroP binding site residues corresponding to Ser-486 and His-488 in LtaS_Lm_ were also conserved, however, only found in LtaS-type but not in primase-like enzymes (Fig. 7*C*). Based on our functional data, which showed that residues Ser-486 and His-488 are required for LTA production, we suggest that the absence of these residues is an important factor contributing to the inability of the LtaP enzyme to produce a PGP polymer.

## DISCUSSION

### 

#### 

##### Model for the Enzyme Reaction Mechanism and LTA Chain Extension of LtaS-type Enzymes

**FIGURE 10.**
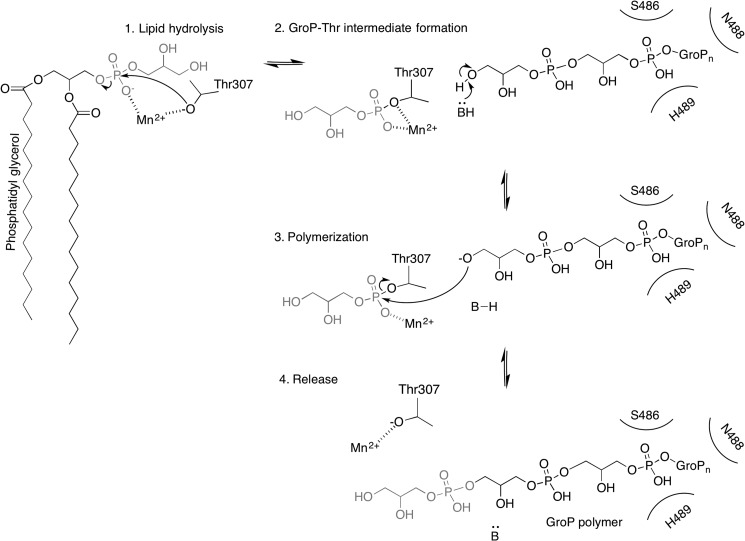
**Proposed reaction mechanism of LtaS_Lm_.** The active site threonine is polarized by the Mn^2+^ ion allowing for a nucleophilic attack of Thr-307 to PG (*1*) generating the Thr-glycerolphosphate intermediate with the elimination of a DAG molecule (2). In our model the penultimate GroP molecule of the incoming GroP chain (GroP*_n_*) would be held in place within the second GroP binding site. The hydroxyl group of the terminal GroP unit will be deprotonated by a base (amino acid residue or water) (*2*) allowing for a nucleophilic attack on the Thr-GroP intermediate to occur (*3*). The product of the reaction, the LTA chain extended by one GroP unit, is released and the cycle completed through the deprotonation of the base of the reaction and the catalytic Thr-307 is repolarized by the metal ion (*4*).

Our new data presented in this study combined with previous results allow us to speculate how the LTA synthesis proceeds. We suggest that the reaction is initiated by nucleophilic attack of Thr-307 to PG resulting in the breakage of the phosphoester bond yielding one molecule of DAG and a covalent GroP-Thr intermediate (Fig. 10). LtaS belongs to the alkaline phosphatase superfamily and arylsulfatase family, in which Ser and Thr residues are often phosphorylated to be activated (39). For this reason it has been postulated that phosphorylation of the catalytic Thr as observed in the *B. subtilis* LtaS structure is required for initiation of the reaction (13, 18). However, we show in the current study that this is not the case for eLtaS_Lm_. Although the active site threonine residue is phosphorylated in the eLtaS_Lm_ structure (Fig. 1), mass spectrometry analysis showed that this phosphorylation is likely an artifact caused by the purification of the protein from *E. coli* extracts as only a very small fraction of the protein obtained from the natural host *L. monocygenes* is phosphorylated (Fig. 3). The threonine phosphorylation is more likely to mimic the covalent GroP-Thr intermediate.

Next, the covalent GroP-Thr intermediate (GroP donor molecule) has to be attached to the incoming LTA chain (GroP acceptor molecule). In this study, we identified a second GroP binding site in the *L. monocytogenes* LtaS enzyme, which consists of residues S486A, N488A, and H489A. A reanalysis of the previously published *S. aureus* and *B. subtilis* eLtaS revealed that this binding site is identical in all three enzymes. It can be speculated that the tip of the LTA chain is bound in a similar manner to the GroP molecule within this second binding site. However, for a transfer reaction to occur, the enzyme would need to undergo a significant conformational change in order for the terminal hydroxyl group to reach the 6.3 Å removed charged active site threonine. Therefore we hypothesize that the trapped GroP molecule represents more likely the penultimate GroP subunit of a growing LTA chain (Figs. 6 and 10). Residues Lys-306 and Tyr-483 were located close to the active center, and could assist the binding of a terminal GroP subunit of an incoming chain by coordinating its phosphate group (Fig. 6). No electron density is observed for the side chain of Lys-306 in both the *Listeria* and *Staphylococcus* eLtaS enzymes, suggesting that the lysine is flexible and therefore could be used for stabilizing the phosphate group of an incoming terminal GroP (Fig. 6). It is of note that both Lys-306 and Tyr-483 are conserved residues among LtaS-type enzymes. In LtaP-type enzymes, where there is no requirement for binding of incoming GroP chains, these residues are replaced with Asn-278 and a range of amino acids at position 457 (Figs. 7*C*).

For the polymerization reaction to occur the proton of the terminal hydroxyl group of the incoming LTA chain must be displaced. No obvious candidate residues can be identified in the vicinity of this terminal GroP or near the bound GroP2. Previous findings showing that the full-length enzyme is required *in vivo* for LTA production highlights a crucial function of the membrane domain for enzyme function (17). One hypothesis is that a residue(s) within the transmembrane domain of the full-length LtaS enzyme could act as a base to remove a proton from the hydroxyl group of the acceptor GroP chain. Based on topology predictions, LtaS_Lm_ has five transmembrane helices and two extracellular loops, which span residues 35 to 48 (extracellular loop 1) and residues 98 to 105 (extracellular loop 2). Strikingly Asp-101 and Phe-102 within the second loop are highly conserved among LtaS-type enzymes but not in LtaP (data not shown) suggesting a possible functional role for these residues; in particular Asp-101 could act as a base required for the polymerization reaction. Once the terminal hydroxyl group is deprotonated it can act as a nucleophile to attack the phosphoester of the bound GroP-Thr assisted by the bound metal (Fig. 10).

**FIGURE 11.**
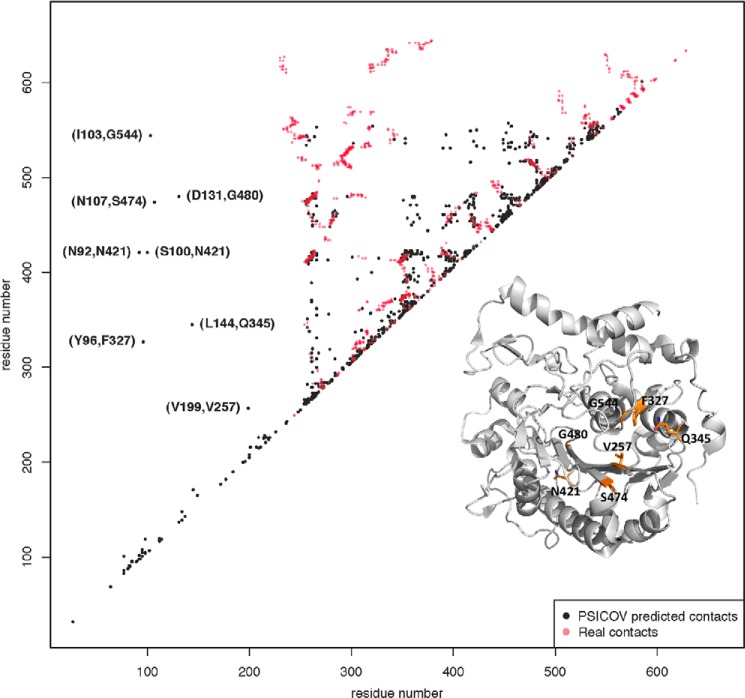
**Experimental and PSICOV-predicted contacts in LtaS_Lm_.** PSICOV-predicted residue contacts using 6943 sequences are shown in *black* and experimentally confirmed contacting amino acids (Cβ-Cβ distance <8 Å) based on the LtaS_Lm_ structure within the extracellular domain are shown in *red*. The eight predicted transmembrane domain extracellular domain contacts are labeled based on the LtaS_Lm_ amino acid numbering. *Inset* shows eLtaS_Lm_ structure with contacting amino acids in the extracellular domain highlighted in *orange*.

To date, no structural information is available for the membrane portion of any of the LTA synthesis enzymes. Previously it has been reported that hybrid proteins, in which the membrane and extracellular domains of two functional proteins are swapped, are non-functional suggesting a specific interaction between the transmembrane and extracellular enzymatic domains (17). If a direct interaction between the two domains is crucial for enzyme function, one might expect interacting amino acids to co-vary within the two domains of LtaS enzymes. To explore this, a new larger alignment was made using 6943 sequences from the non-redundant database. Residue contacts were predicted using PSICOV and plotted alongside experimentally confirmed contacting amino acids based on the eLtaS_Lm_ structure (Fig. 11). Using this analysis, several residues within the transmembrane region were predicted to be in contact with amino acid residues within the extracellular domain (primarily located in proximity of the active site or at the back of the molecule), supporting the notion of a physical interaction between the transmembrane and extracellular domain.

The LtaP_Lm_ and LtaS_Lm_ structures determined as part of this study provide information on the molecular basis for the restricted enzyme activity and inability of the LtaP_Lm_ enzyme to polymerize LTA chains. Specifically, our work revealed that LtaP_Lm_ has a smaller active site cavity, lacks a second GroP binding site, and that two conserved loop insertions results in subtle alterations to surface cavities. These data allowed us to propose a model on how the incoming LTA chain could bind during the chain extension step. Supported by bioinformatics analyses, we further suggest that a crucial catalytic residue for activating the GroP acceptor chain might be located within the transmembrane domain. To confirm this and to understand the functional significance of highly conserved amino acids within the extracellular loops or the conserved aspartic acid residues with the fourth transmembrane helix will require further studies and in particular a structural investigation on the full-length enzyme.

LTA synthesis enzymes are currently being actively pursued as target proteins for the development of novel antibiotics and recently, the first LtaS enzyme inhibitor was identified (4). Based on our findings, we would suggest that future structure-based design of LTA synthesis enzyme inhibitors should be extended to include the second GroP binding site. We envisage that targeting this binding site may offer a better chance of obtaining LtaS-specific inhibitors and decrease the possibility of obtaining compounds that are cross-reactive toward members of the same protein family such as mammalian alkaline phosphatases. Expanding the chemical landscape search to a larger enzyme area might increase the chances of discovering new enzyme-specific inhibitors, which could be used to treat infections caused by important Gram-positive human pathogens.

## Supplementary Material

Supplemental Data

## Footnotes

3 The abbreviations used are:

LTA: lipoteichoic acid
PGP: polyphosphatidylglycerol phosphate
DAG: diacylglycerol
PG: phosphatidylglycerolphosphate
Ptd: phosphatidyl
GroP: glycerolphosphate
PDB: Protein Data Bank
NBD: 7-nitrobenz-2-oxa-1,3-diazole-4-yl.
