# Supplementary material for: Structural and Mechanistic Insight into the Listeria monocytogenes Two-enzyme Lipoteichoic Acid Synthesis System
Source: J Biol Chem. 2014 Aug 15;289(41):28054–69. doi: 10.1074/jbc.M114.590570 (PMC4192460; doi:10.1074/jbc.M114.590570)
Supplement: Supplemental Data [file supp_289_41_28054__index.html]

Structural and Mechanistic Insight into the Listeria monocytogenes Two-Enzyme Lipoteichoic Acid Synthesis System — Structural and Mechanistic Insight into the Listeria monocytogenes Two-enzyme Lipoteichoic Acid Synthesis System — Structural Analysis of LTA Synthesis Enzymes — Supplemental Data 

# Structural and Mechanistic Insight into the *Listeria monocytogenes* Two-enzyme Lipoteichoic Acid Synthesis System

## Supplemental Data

**Files in this Data Supplement:**

- Supplemental Table S1 (.pdf, 197 KB) - Organisms and RefSeq accession numbers for LTA primase-like and LTA synthase-like sequences.
