# Supplementary material for: Structural and Mechanistic Insight into the Listeria monocytogenes Two-enzyme Lipoteichoic Acid Synthesis System
Source: J Biol Chem. 2014 Aug 15;289(41):28054–69. doi: 10.1074/jbc.M114.590570 (PMC4192460; doi:10.1074/jbc.M114.590570)
Supplement: Supplemental Data [file supp_M114.590570_jbc.M114.590570-1.pdf]

SUPPLEMENTAL TABLE S1: Organisms and RefSeq accession numbers for LTA primase-like sequences shown in red and LTA synthase-like sequences shown in green if the same bacterial stain also contains an LtaP-like enzyme or in in black if the bacterial strain only contains LtaS-like enzymes.

Strain	RefSeq accession	Synthase present Yes/NO
Bacillus cereus subsp. cytotoxis NVH 391-98	YP_001374421.1	YES
Brevibacillus laterosporus LMG 15441	ZP_08641210.1	NO
Listeria grayi DSM 20601	ZP_07054112.1	YES
Listeria innocua Clip11262	NP_469990.1	YES
Listeria ivanovii FSL F6-596	ZP_07872889.1	YES
Listeria marthii FSL S4-120	ZP_07869935.1	YES
Listeria monocytogenes 08-5578	YP_003412836.1	YES
Listeria monocytogenes 08-5923	YP_003415881.1	YES
Listeria monocytogenes 10403S	ZP_05235023.1	YES
Listeria monocytogenes Clip81459	YP_002757375.1	YES
Listeria monocytogenes EGD-e	NP_464171.1	YES
Listeria monocytogenes F6900	ZP_05267348.2	YES
Listeria monocytogenes Finland 1988	ZP_03667074.1	YES
Listeria monocytogenes FSL J1-194	ZP_05229081.2	YES
Listeria monocytogenes FSL J2-064	ZP_05276090.1	YES
Listeria monocytogenes FSL N1-017	ZP_07074442.1	YES
Listeria monocytogenes FSL N3-165	ZP_05232976.2	YES
Listeria monocytogenes FSL R2-503	ZP_05242503.2	YES
Listeria monocytogenes FSL R2-561	ZP_03670361.1	YES
Listeria monocytogenes HCC23	YP_002350942.1	YES
Listeria monocytogenes HPB2262	ZP_05265439.2	YES
Listeria monocytogenes J0161	ZP_05260046.1	YES
Listeria monocytogenes J2818	ZP_05261336.2	YES
Listeria monocytogenes LO28	ZP_05300700.1	NO
Listeria monocytogenes serotype 4b str. F2365	YP_013279.1	YES
Listeria monocytogenes str. 1/2a F6854	ZP_00233907.1	YES
Listeria monocytogenes str. 4b H7858	ZP_00229399.1	YES
Listeria seeligeri serovar 1/2b str. SLCC3954	YP_003463799.1	YES
Listeria welshimeri serovar 6b str. SLCC5334	YP_848812.1	YES
Paenibacillus curdlanolyticus YK9	ZP_07386669.1	NO
Paenibacillus curdlanolyticus YK9	ZP_07388183.1	NO
Paenibacillus sp. HGF5	ZP_08278017.1	YES
Paenibacillus sp. HGF5	ZP_08278918.1	YES
Paenibacillus sp. HGF7	ZP_08508213.1	NO
Paenibacillus sp. JDR-2	YP_003009960.1	NO
Paenibacillus sp. JDR-2	YP_003013709.1	NO
Paenibacillus sp. JDR-2	YP_003014584.1	NO
Paenibacillus sp. JDR-2	YP_003014692.1	NO
Paenibacillus sp. oral taxon 786 str. D14	ZP_04851738.1	NO
Paenibacillus sp. oral taxon 786 str. D14	ZP_04851934.1	NO
Paenibacillus sp. Y412MC10	YP_003242571.1	YES
Paenibacillus sp. Y412MC10	YP_003246020.1	YES
Paenibacillus vortex V453	ZP_07897126.1	YES
Paenibacillus vortex V453	ZP_07899822.1	YES
Planococcus donghaensis MPA1U2	ZP_08095601.1	YES
Planococcus donghaensis MPA1U2	ZP_08096155.1	YES
Thermotoga maritima MSB8	NP_229503.1	NO
Thermotoga naphthophila RKU-10	YP_003346604.1	NO

Thermotoga petrophila RKU-1	YP_001244593.1	NO
Thermotoga sp. RQ2	YP_001739159.1	NO
Acetivibrio cellulolyticus CD2	ZP_07325283.1	
Acetivibrio cellulolyticus CD2	ZP_07328110.1	
Aminomonas paucivorans DSM 12260	ZP_07740656.1	
Anoxybacillus flavithermus WK1	YP_002315259.1	
Bacillus amyloliquefaciens DSM 7	YP_003919292.1	
Bacillus amyloliquefaciens DSM 7	YP_003919336.1	
Bacillus amyloliquefaciens FZB42	YP_001420367.1	
Bacillus amyloliquefaciens FZB42	YP_001420407.1	
Bacillus amyloliquefaciens FZB42	YP_001422610.1	
Bacillus anthracis CI	YP_003791267.1	
Bacillus anthracis CI	YP_003792666.1	
Bacillus anthracis CI	YP_003794906.1	
Bacillus anthracis str. 'Ames Ancestor'	YP_018060.1	
Bacillus anthracis str. 'Ames Ancestor'	YP_019588.1	
Bacillus anthracis str. 'Ames Ancestor'	YP_020532.1	
Bacillus anthracis str. 'Ames Ancestor'	YP_022133.1	
Bacillus anthracis str. A0174	ZP_02932633.1	
Bacillus anthracis str. A0174	ZP_02934314.1	
Bacillus anthracis str. A0174	ZP_02934695.1	
Bacillus anthracis str. A0193	ZP_02396158.1	
Bacillus anthracis str. A0193	ZP_02397491.1	
Bacillus anthracis str. A0193	ZP_02398098.1	
Bacillus anthracis str. A0248	YP_002865928.1	
Bacillus anthracis str. A0248	YP_002867196.1	
Bacillus anthracis str. A0248	YP_002868001.1	
Bacillus anthracis str. A0248	YP_002869449.1	
Bacillus anthracis str. A0389	ZP_02894944.1	
Bacillus anthracis str. A0389	ZP_02896832.1	
Bacillus anthracis str. A0389	ZP_02897220.1	
Bacillus anthracis str. A0442	ZP_02391019.1	
Bacillus anthracis str. A0442	ZP_02392765.1	
Bacillus anthracis str. A0442	ZP_02394124.1	
Bacillus anthracis str. A0465	ZP_02876690.1	
Bacillus anthracis str. A0465	ZP_02878001.1	
Bacillus anthracis str. A0465	ZP_02879091.1	
Bacillus anthracis str. A0488	ZP_02213764.1	
Bacillus anthracis str. A0488	ZP_02214599.1	
Bacillus anthracis str. A0488	ZP_02215059.1	
Bacillus anthracis str. A0488	ZP_02217022.1	
Bacillus anthracis str. A1055	ZP_05183842.1	
Bacillus anthracis str. A1055	ZP_05184698.1	
Bacillus anthracis str. A1055	ZP_05186125.1	
Bacillus anthracis str. A1055	ZP_05187866.1	
Bacillus anthracis str. A2012	ZP_00390161.1	
Bacillus anthracis str. A2012	ZP_00391742.1	
Bacillus anthracis str. A2012	ZP_00393179.1	
Bacillus anthracis str. A2012	ZP_00394046.1	
Bacillus anthracis str. Ames	NP_843892.1	
Bacillus anthracis str. Ames	NP_845283.1	
Bacillus anthracis str. Ames	NP_846142.1	
Bacillus anthracis str. Ames	NP_847636.1	
Bacillus anthracis str. Australia 94	ZP_05210985.1	

Bacillus anthracis str. Australia 94	ZP_05211414.1
Bacillus anthracis str. Australia 94	ZP_05211628.1
Bacillus anthracis str. Australia 94	ZP_05212815.1
Bacillus anthracis str. CDC 684	YP_002813342.1
Bacillus anthracis str. CDC 684	YP_002814258.1
Bacillus anthracis str. CDC 684	YP_002815744.1
Bacillus anthracis str. CDC 684	YP_002818000.1
Bacillus anthracis str. CNEVA-9066	ZP_05146281.1
Bacillus anthracis str. CNEVA-9066	ZP_05147392.1
Bacillus anthracis str. CNEVA-9066	ZP_05149363.1
Bacillus anthracis str. CNEVA-9066	ZP_05150204.1
Bacillus anthracis str. Kruger B	ZP_05197369.1
Bacillus anthracis str. Kruger B	ZP_05198163.1
Bacillus anthracis str. Kruger B	ZP_05200552.1
Bacillus anthracis str. Kruger B	ZP_05202042.1
Bacillus anthracis str. Sterne	YP_027596.1
Bacillus anthracis str. Sterne	YP_028995.1
Bacillus anthracis str. Sterne	YP_029861.1
Bacillus anthracis str. Sterne	YP_031320.1
Bacillus anthracis str. Vollum	ZP_05203110.1
Bacillus anthracis str. Vollum	ZP_05204311.1
Bacillus anthracis str. Vollum	ZP_05205898.1
Bacillus anthracis str. Vollum	ZP_05207697.1
Bacillus anthracis str. Western North America USA6153	ZP_05191550.1
Bacillus anthracis str. Western North America USA6153	ZP_05193505.1
Bacillus anthracis str. Western North America USA6153	ZP_05194740.1
Bacillus anthracis str. Western North America USA6153	ZP_05195662.1
Bacillus anthracis Tsiankovskii-I	ZP_03017703.1
Bacillus anthracis Tsiankovskii-I	ZP_03020710.1
Bacillus anthracis Tsiankovskii-I	ZP_03021027.1
Bacillus anthracis Tsiankovskii-I	ZP_03021313.1
Bacillus atrophaeus 1942	YP_003972134.1
Bacillus atrophaeus 1942	YP_003972181.1
Bacillus atrophaeus 1942	YP_003974009.1
Bacillus atrophaeus 1942	YP_003974777.1
Bacillus cellulosilyticus DSM 2522	YP_004094405.1
Bacillus cellulosilyticus DSM 2522	YP_004096437.1
Bacillus cereus 03BB102	YP_002748758.1
Bacillus cereus 03BB102	YP_002750290.1
Bacillus cereus 03BB102	YP_002751123.1
Bacillus cereus 03BB102	YP_002752580.1
Bacillus cereus 03BB108	ZP_03110352.1
Bacillus cereus 03BB108	ZP_03111782.1
Bacillus cereus 03BB108	ZP_03112809.1
Bacillus cereus 03BB108	ZP_03114537.1
Bacillus cereus 172560W	ZP_04305296.1
Bacillus cereus 172560W	ZP_04306578.1
Bacillus cereus 172560W	ZP_04307386.1
Bacillus cereus 172560W	ZP_04308818.1
Bacillus cereus 95/8201	ZP_04250298.1
Bacillus cereus 95/8201	ZP_04251698.1
Bacillus cereus 95/8201	ZP_04252482.1
Bacillus cereus 95/8201	ZP_04253919.1
Bacillus cereus AH1134	ZP_03228734.1

Bacillus cereus AH1134	ZP_03231431.1
Bacillus cereus AH1134	ZP_03232669.1
Bacillus cereus AH1134	ZP_03233556.1
Bacillus cereus AH1271	ZP_04185288.1
Bacillus cereus AH1271	ZP_04186652.1
Bacillus cereus AH1271	ZP_04187395.1
Bacillus cereus AH1271	ZP_04188807.1
Bacillus cereus AH1272	ZP_04179494.1
Bacillus cereus AH1272	ZP_04180888.1
Bacillus cereus AH1272	ZP_04181622.1
Bacillus cereus AH1272	ZP_04183018.1
Bacillus cereus AH1273	ZP_04173702.1
Bacillus cereus AH1273	ZP_04175125.1
Bacillus cereus AH1273	ZP_04175797.1
Bacillus cereus AH1273	ZP_04177202.1
Bacillus cereus AH187	YP_002337542.1
Bacillus cereus AH187	YP_002338943.1
Bacillus cereus AH187	YP_002339746.1
Bacillus cereus AH187	YP_002341293.1
Bacillus cereus AH603	ZP_04196540.1
Bacillus cereus AH603	ZP_04198713.1
Bacillus cereus AH603	ZP_04200190.1
Bacillus cereus AH621	ZP_04294115.1
Bacillus cereus AH621	ZP_04296227.1
Bacillus cereus AH621	ZP_04297635.1
Bacillus cereus AH676	ZP_04190980.1
Bacillus cereus AH676	ZP_04192290.1
Bacillus cereus AH676	ZP_04193040.1
Bacillus cereus AH676	ZP_04194449.1
Bacillus cereus AH820	YP_002450459.1
Bacillus cereus AH820	YP_002451896.1
Bacillus cereus AH820	YP_002452726.1
Bacillus cereus AH820	YP_002454241.1
Bacillus cereus ATCC 10876	ZP_04316613.1
Bacillus cereus ATCC 10876	ZP_04318028.1
Bacillus cereus ATCC 10876	ZP_04318861.1
Bacillus cereus ATCC 10876	ZP_04320425.1
Bacillus cereus ATCC 10987	NP_977859.1
Bacillus cereus ATCC 10987	NP_979290.1
Bacillus cereus ATCC 10987	NP_980094.1
Bacillus cereus ATCC 10987	NP_981640.1
Bacillus cereus ATCC 14579	NP_831197.1
Bacillus cereus ATCC 14579	NP_832680.1
Bacillus cereus ATCC 14579	NP_833488.1
Bacillus cereus ATCC 14579	NP_834895.1
Bacillus cereus ATCC 4342	ZP_04283198.1
Bacillus cereus ATCC 4342	ZP_04284618.1
Bacillus cereus ATCC 4342	ZP_04285421.1
Bacillus cereus ATCC 4342	ZP_04286852.1
Bacillus cereus B4264	YP_002366199.1
Bacillus cereus B4264	YP_002367659.1
Bacillus cereus B4264	YP_002368568.1
Bacillus cereus B4264	YP_002370007.1
Bacillus cereus BDRD-Cer4	ZP_04255844.1

Bacillus cereus BDRD-Cer4	ZP_04257258.1
Bacillus cereus BDRD-Cer4	ZP_04258015.1
Bacillus cereus BDRD-Cer4	ZP_04259434.1
Bacillus cereus BDRD-ST196	ZP_04261177.1
Bacillus cereus BDRD-ST196	ZP_04262635.1
Bacillus cereus BDRD-ST196	ZP_04263382.1
Bacillus cereus BDRD-ST196	ZP_04264799.1
Bacillus cereus BDRD-ST24	ZP_04272532.1
Bacillus cereus BDRD-ST24	ZP_04273898.1
Bacillus cereus BDRD-ST24	ZP_04274708.1
Bacillus cereus BDRD-ST24	ZP_04276105.1
Bacillus cereus BDRD-ST26	ZP_04266803.1
Bacillus cereus BDRD-ST26	ZP_04268148.1
Bacillus cereus BDRD-ST26	ZP_04268952.1
Bacillus cereus BDRD-ST26	ZP_04270491.1
Bacillus cereus BGSC 6E1	ZP_04310932.1
Bacillus cereus BGSC 6E1	ZP_04312338.1
Bacillus cereus BGSC 6E1	ZP_04313166.1
Bacillus cereus BGSC 6E1	ZP_04314592.1
Bacillus cereus E33L	YP_082900.1
Bacillus cereus E33L	YP_084253.1
Bacillus cereus E33L	YP_085099.1
Bacillus cereus E33L	YP_086498.1
Bacillus cereus F65185	ZP_04202361.1
Bacillus cereus F65185	ZP_04203656.1
Bacillus cereus F65185	ZP_04204485.1
Bacillus cereus F65185	ZP_04205894.1
Bacillus cereus G9241	ZP_00237335.1
Bacillus cereus G9241	ZP_00238965.1
Bacillus cereus G9241	ZP_00239448.1
Bacillus cereus G9241	ZP_00241253.1
Bacillus cereus G9842	YP_002444859.1
Bacillus cereus G9842	YP_002446428.1
Bacillus cereus G9842	YP_002447277.1
Bacillus cereus G9842	YP_002448761.1
Bacillus cereus H3081.97	ZP_03234457.1
Bacillus cereus H3081.97	ZP_03238538.1
Bacillus cereus H3081.97	ZP_03238793.1
Bacillus cereus m1293	ZP_04322477.1
Bacillus cereus m1293	ZP_04323856.1
Bacillus cereus m1293	ZP_04324585.1
Bacillus cereus m1293	ZP_04326012.1
Bacillus cereus m1550	ZP_04277947.1
Bacillus cereus m1550	ZP_04279358.1
Bacillus cereus m1550	ZP_04280162.1
Bacillus cereus m1550	ZP_04281568.1
Bacillus cereus MM3	ZP_04299720.1
Bacillus cereus MM3	ZP_04301137.1
Bacillus cereus MM3	ZP_04303399.1
Bacillus cereus NVH0597-99	ZP_03105790.1
Bacillus cereus NVH0597-99	ZP_03106696.1
Bacillus cereus NVH0597-99	ZP_03109473.1
Bacillus cereus NVH0597-99	ZP_03109504.1
Bacillus cereus Q1	YP_002529210.1

Bacillus cereus Q1	YP_002530494.1
Bacillus cereus Q1	YP_002531267.1
Bacillus cereus Q1	YP_002532749.1
Bacillus cereus R309803	ZP_04288463.1
Bacillus cereus R309803	ZP_04289783.1
Bacillus cereus R309803	ZP_04290651.1
Bacillus cereus R309803	ZP_04292108.1
Bacillus cereus Rock1-15	ZP_04238575.1
Bacillus cereus Rock1-15	ZP_04239962.1
Bacillus cereus Rock1-15	ZP_04240792.1
Bacillus cereus Rock1-15	ZP_04242176.1
Bacillus cereus Rock1-3	ZP_04244374.1
Bacillus cereus Rock1-3	ZP_04245850.1
Bacillus cereus Rock1-3	ZP_04246629.1
Bacillus cereus Rock1-3	ZP_04248075.1
Bacillus cereus Rock3-28	ZP_04232831.1
Bacillus cereus Rock3-28	ZP_04234230.1
Bacillus cereus Rock3-28	ZP_04234998.1
Bacillus cereus Rock3-28	ZP_04236463.1
Bacillus cereus Rock3-29	ZP_04226983.1
Bacillus cereus Rock3-29	ZP_04228419.1
Bacillus cereus Rock3-29	ZP_04229181.1
Bacillus cereus Rock3-29	ZP_04230586.1
Bacillus cereus Rock3-42	ZP_04221711.1
Bacillus cereus Rock3-42	ZP_04223131.1
Bacillus cereus Rock3-42	ZP_04223935.1
Bacillus cereus Rock3-42	ZP_04225408.1
Bacillus cereus Rock3-44	ZP_04216788.1
Bacillus cereus Rock3-44	ZP_04217929.1
Bacillus cereus Rock3-44	ZP_04219831.1
Bacillus cereus Rock4-18	ZP_04207727.1
Bacillus cereus Rock4-18	ZP_04209407.1
Bacillus cereus Rock4-2	ZP_04211255.1
Bacillus cereus Rock4-2	ZP_04212650.1
Bacillus cereus Rock4-2	ZP_04213531.1
Bacillus cereus Rock4-2	ZP_04214906.1
Bacillus cereus SJ1	ZP_07055550.1
Bacillus cereus SJ1	ZP_07056274.1
Bacillus cereus subsp. cytotoxis NVH 391-98	YP_001374459.1
Bacillus cereus subsp. cytotoxis NVH 391-98	YP_001375280.1
Bacillus cereus subsp. cytotoxis NVH 391-98	YP_001376952.1
Bacillus cereus W	ZP_03099811.1
Bacillus cereus W	ZP_03100464.1
Bacillus cereus W	ZP_03101446.1
Bacillus cereus W	ZP_03102721.1
Bacillus clausii KSM-K16	YP_174302.1
Bacillus clausii KSM-K16	YP_176712.1
Bacillus clausii KSM-K16	YP_176787.1
Bacillus coagulans 2-6	YP_004568714.1
Bacillus coagulans 36D1	ZP_04433360.1
Bacillus coahuilensis m4-4	ZP_03226815.1
Bacillus licheniformis ATCC 14580	YP_078018.1
Bacillus licheniformis ATCC 14580	YP_079810.1
Bacillus licheniformis ATCC 14580	YP_080619.1

Bacillus licheniformis ATCC 14580	YP_081231.1
Bacillus licheniformis ATCC 14580	YP_090421.1
Bacillus licheniformis ATCC 14580	YP_092225.1
Bacillus licheniformis ATCC 14580	YP_093046.1
Bacillus licheniformis ATCC 14580	YP_093665.1
Bacillus megaterium DSM 319	YP_003596057.1
Bacillus megaterium DSM 319	YP_003597184.1
Bacillus megaterium DSM 319	YP_003599111.1
Bacillus megaterium QM B1551	YP_003561311.1
Bacillus megaterium QM B1551	YP_003562488.1
Bacillus megaterium QM B1551	YP_003564392.1
Bacillus mycoides DSM 2048	ZP_04168018.1
Bacillus mycoides DSM 2048	ZP_04169377.1
Bacillus mycoides DSM 2048	ZP_04170112.1
Bacillus mycoides DSM 2048	ZP_04171519.1
Bacillus mycoides Rock1-4	ZP_04162077.1
Bacillus mycoides Rock1-4	ZP_04163096.1
Bacillus mycoides Rock1-4	ZP_04166639.1
Bacillus mycoides Rock3-17	ZP_04156266.1
Bacillus mycoides Rock3-17	ZP_04157449.1
Bacillus mycoides Rock3-17	ZP_04159546.1
Bacillus pseudomycoides DSM 12442	ZP_04150492.1
Bacillus pseudomycoides DSM 12442	ZP_04151687.1
Bacillus pseudomycoides DSM 12442	ZP_04154815.1
Bacillus pumilus ATCC 7061	ZP_03052910.1
Bacillus pumilus ATCC 7061	ZP_03054152.1
Bacillus pumilus ATCC 7061	ZP_03054773.1
Bacillus pumilus ATCC 7061	ZP_03056324.1
Bacillus pumilus SAFR-032	YP_001485971.1
Bacillus pumilus SAFR-032	YP_001486083.1
Bacillus pumilus SAFR-032	YP_001487443.1
Bacillus pumilus SAFR-032	YP_001488215.1
Bacillus selenitireducens MLS10	YP_003701292.1
Bacillus sp. 2_A_57_CT2	ZP_08003405.1
Bacillus sp. 2_A_57_CT2	ZP_08006844.1
Bacillus sp. 2_A_57_CT2	ZP_08007352.1
Bacillus sp. 2_A_57_CT2	ZP_08008125.1
Bacillus sp. 2_A_57_CT2	ZP_08008487.1
Bacillus sp. B14905	ZP_01725672.1
Bacillus sp. B14905	ZP_01726201.1
Bacillus sp. BT1B_CT2	ZP_07999256.1
Bacillus sp. BT1B_CT2	ZP_08001220.1
Bacillus sp. BT1B_CT2	ZP_08001964.1
Bacillus sp. BT1B_CT2	ZP_08002533.1
Bacillus sp. m3-13	ZP_07708925.1
Bacillus sp. NRRL B-14911	ZP_01169085.1
Bacillus sp. NRRL B-14911	ZP_01169135.1
Bacillus sp. NRRL B-14911	ZP_01172073.1
Bacillus sp. NRRL B-14911	ZP_01173893.1
Bacillus sp. SG-1	ZP_01859102.1
Bacillus sp. SG-1	ZP_01861366.1
Bacillus subtilis BSn5	YP_004204256.1
Bacillus subtilis BSn5	YP_004205173.1
Bacillus subtilis BSn5	YP_004206743.1

Bacillus subtilis BSn5	YP_004206787.1
Bacillus subtilis subsp. spizizenii ATCC 6633	ZP_06873654.1
Bacillus subtilis subsp. spizizenii ATCC 6633	ZP_06874285.1
Bacillus subtilis subsp. spizizenii ATCC 6633	ZP_06874329.1
Bacillus subtilis subsp. spizizenii ATCC 6633	ZP_06875811.1
Bacillus subtilis subsp. spizizenii str. W23	YP_003865104.1
Bacillus subtilis subsp. spizizenii str. W23	YP_003865148.1
Bacillus subtilis subsp. spizizenii str. W23	YP_003866809.1
Bacillus subtilis subsp. spizizenii str. W23	YP_003867613.1
Bacillus subtilis subsp. subtilis str. 168	NP_388607.2
Bacillus subtilis subsp. subtilis str. 168	NP_388652.1
Bacillus subtilis subsp. subtilis str. 168	NP_390364.2
Bacillus subtilis subsp. subtilis str. 168	NP_391216.1
Bacillus subtilis subsp. subtilis str. 168	ZP_03590410.1
Bacillus subtilis subsp. subtilis str. 168	ZP_03590456.1
Bacillus subtilis subsp. subtilis str. 168	ZP_03592255.1
Bacillus subtilis subsp. subtilis str. 168	ZP_03593133.1
Bacillus subtilis subsp. subtilis str. JH642	ZP_03599106.1
Bacillus subtilis subsp. subtilis str. JH642	ZP_03599151.1
Bacillus subtilis subsp. subtilis str. JH642	ZP_03600948.1
Bacillus subtilis subsp. subtilis str. JH642	ZP_03601823.1
Bacillus subtilis subsp. subtilis str. NCIB 3610	ZP_03594693.1
Bacillus subtilis subsp. subtilis str. NCIB 3610	ZP_03594738.1
Bacillus subtilis subsp. subtilis str. NCIB 3610	ZP_03596536.1
Bacillus subtilis subsp. subtilis str. NCIB 3610	ZP_03597418.1
Bacillus subtilis subsp. subtilis str. SMY	ZP_03603380.1
Bacillus subtilis subsp. subtilis str. SMY	ZP_03603425.1
Bacillus subtilis subsp. subtilis str. SMY	ZP_03605225.1
Bacillus subtilis subsp. subtilis str. SMY	ZP_03606107.1
Bacillus thuringiensis BMB171	YP_003663790.1
Bacillus thuringiensis BMB171	YP_003665171.1
Bacillus thuringiensis BMB171	YP_003665965.1
Bacillus thuringiensis BMB171	YP_003667355.1
Bacillus thuringiensis Bt407	ZP_04138500.1
Bacillus thuringiensis Bt407	ZP_04139868.1
Bacillus thuringiensis Bt407	ZP_04140630.1
Bacillus thuringiensis Bt407	ZP_04142231.1
Bacillus thuringiensis IBL 200	ZP_04071010.1
Bacillus thuringiensis IBL 200	ZP_04072535.1
Bacillus thuringiensis IBL 200	ZP_04073412.1
Bacillus thuringiensis IBL 200	ZP_04074874.1
Bacillus thuringiensis IBL 4222	ZP_04064331.1
Bacillus thuringiensis IBL 4222	ZP_04065663.1
Bacillus thuringiensis IBL 4222	ZP_04066429.1
Bacillus thuringiensis IBL 4222	ZP_04067824.1
Bacillus thuringiensis serovar andalousiensis BGSC 4AW1	ZP_04095671.1
Bacillus thuringiensis serovar andalousiensis BGSC 4AW1	ZP_04097040.1
Bacillus thuringiensis serovar andalousiensis BGSC 4AW1	ZP_04097891.1
Bacillus thuringiensis serovar andalousiensis BGSC 4AW1	ZP_04099315.1
Bacillus thuringiensis serovar berliner ATCC 10792	ZP_04101235.1
Bacillus thuringiensis serovar berliner ATCC 10792	ZP_04102633.1
Bacillus thuringiensis serovar berliner ATCC 10792	ZP_04103403.1
Bacillus thuringiensis serovar berliner ATCC 10792	ZP_04104907.1
Bacillus thuringiensis serovar huazhongensis BGSC 4BD1	ZP_04083575.1

Bacillus thuringiensis serovar huazhongensis BGSC 4BD1	ZP_04084921.1
Bacillus thuringiensis serovar huazhongensis BGSC 4BD1	ZP_04085781.1
Bacillus thuringiensis serovar huazhongensis BGSC 4BD1	ZP_04087240.1
Bacillus thuringiensis serovar israelensis ATCC 35646	ZP_00740466.1
Bacillus thuringiensis serovar israelensis ATCC 35646	ZP_00744071.1
Bacillus thuringiensis serovar konkukian str. 97-27	YP_035634.1
Bacillus thuringiensis serovar konkukian str. 97-27	YP_037012.1
Bacillus thuringiensis serovar konkukian str. 97-27	YP_037825.1
Bacillus thuringiensis serovar konkukian str. 97-27	YP_039222.1
Bacillus thuringiensis serovar kurstaki str. T03a001	ZP_04113993.1
Bacillus thuringiensis serovar kurstaki str. T03a001	ZP_04115324.1
Bacillus thuringiensis serovar kurstaki str. T03a001	ZP_04116071.1
Bacillus thuringiensis serovar kurstaki str. T03a001	ZP_04117481.1
Bacillus thuringiensis serovar monterrey BGSC 4AJ1	ZP_04107475.1
Bacillus thuringiensis serovar monterrey BGSC 4AJ1	ZP_04108864.1
Bacillus thuringiensis serovar monterrey BGSC 4AJ1	ZP_04109715.1
Bacillus thuringiensis serovar monterrey BGSC 4AJ1	ZP_04111224.1
Bacillus thuringiensis serovar pakistani str. T13001	ZP_04119534.1
Bacillus thuringiensis serovar pakistani str. T13001	ZP_04120857.1
Bacillus thuringiensis serovar pakistani str. T13001	ZP_04121637.1
Bacillus thuringiensis serovar pakistani str. T13001	ZP_04123098.1
Bacillus thuringiensis serovar pondicheriensis BGSC 4BA1	ZP_04089619.1
Bacillus thuringiensis serovar pondicheriensis BGSC 4BA1	ZP_04091034.1
Bacillus thuringiensis serovar pondicheriensis BGSC 4BA1	ZP_04091846.1
Bacillus thuringiensis serovar pondicheriensis BGSC 4BA1	ZP_04093241.1
Bacillus thuringiensis serovar pulsiensis BGSC 4CC1	ZP_04077711.1
Bacillus thuringiensis serovar pulsiensis BGSC 4CC1	ZP_04079125.1
Bacillus thuringiensis serovar pulsiensis BGSC 4CC1	ZP_04079964.1
Bacillus thuringiensis serovar pulsiensis BGSC 4CC1	ZP_04081376.1
Bacillus thuringiensis serovar sotto str. T04001	ZP_04125596.1
Bacillus thuringiensis serovar sotto str. T04001	ZP_04126956.1
Bacillus thuringiensis serovar sotto str. T04001	ZP_04127737.1
Bacillus thuringiensis serovar sotto str. T04001	ZP_04129345.1
Bacillus thuringiensis serovar thuringiensis str. T01001	ZP_04132135.1
Bacillus thuringiensis serovar thuringiensis str. T01001	ZP_04133550.1
Bacillus thuringiensis serovar thuringiensis str. T01001	ZP_04134341.1
Bacillus thuringiensis serovar thuringiensis str. T01001	ZP_04135856.1
Bacillus thuringiensis serovar tochigiensis BGSC 4Y1	ZP_04144770.1
Bacillus thuringiensis serovar tochigiensis BGSC 4Y1	ZP_04146185.1
Bacillus thuringiensis serovar tochigiensis BGSC 4Y1	ZP_04147030.1
Bacillus thuringiensis serovar tochigiensis BGSC 4Y1	ZP_04148545.1
Bacillus thuringiensis str. Al Hakam	YP_894129.1
Bacillus thuringiensis str. Al Hakam	YP_895425.1
Bacillus thuringiensis str. Al Hakam	YP_896136.1
Bacillus thuringiensis str. Al Hakam	YP_897422.1
Bacillus weihenstephanensis KBAB4	YP_001644215.1
Bacillus weihenstephanensis KBAB4	YP_001645569.1
Bacillus weihenstephanensis KBAB4	YP_001646336.1
Bacillus weihenstephanensis KBAB4	YP_001647798.1
Carnobacterium sp. 17-4	YP_004375358.1
Carnobacterium sp. AT7	ZP_02183874.1
Clostridium acetobutylicum ATCC 824	NP_348062.1
Clostridium acetobutylicum ATCC 824	NP_349045.1
Clostridium acetobutylicum DSM 1731	YP_004636102.1

Clostridium acetobutylicum DSM 1731	YP_004637094.1
Clostridium beijerinckii NCIMB 8052	YP_001307516.1
Clostridium botulinum A str. ATCC 19397	YP_001382982.1
Clostridium botulinum A str. ATCC 19397	YP_001385126.1
Clostridium botulinum A str. ATCC 3502	YP_001253135.1
Clostridium botulinum A str. ATCC 3502	YP_001255360.1
Clostridium botulinum A str. Hall	YP_001386528.1
Clostridium botulinum A str. Hall	YP_001388595.1
Clostridium botulinum A2 str. Kyoto	YP_002802939.1
Clostridium botulinum A2 str. Kyoto	YP_002805386.1
Clostridium botulinum A3 str. Loch Maree	YP_001785936.1
Clostridium botulinum A3 str. Loch Maree	YP_001788187.1
Clostridium botulinum B1 str. Okra	YP_001780233.1
Clostridium botulinum B1 str. Okra	YP_001782498.1
Clostridium botulinum Ba4 str. 657	YP_002861482.1
Clostridium botulinum Ba4 str. 657	YP_002863871.1
Clostridium botulinum Bf	ZP_02616211.1
Clostridium botulinum Bf	ZP_02617954.1
Clostridium botulinum F str. Langeland	YP_001389958.1
Clostridium botulinum F str. Langeland	YP_001392142.1
Clostridium botulinum NCTC 2916	ZP_02612629.1
Clostridium botulinum NCTC 2916	ZP_02613960.1
Clostridium carboxidivorans P7	ZP_05391343.1
Clostridium carboxidivorans P7	ZP_05395046.1
Clostridium carboxidivorans P7	ZP_06855904.1
Clostridium carboxidivorans P7	ZP_06856512.1
Clostridium kluyveri DSM 555	YP_001395731.1
Clostridium kluyveri NBRC 12016	YP_002472533.1
Clostridium ljungdahlii DSM 13528	YP_003779047.1
Clostridium ljungdahlii DSM 13528	YP_003780668.1
Clostridium sporogenes ATCC 15579	ZP_02994161.1
Clostridium sporogenes ATCC 15579	ZP_02995030.1
Clostridium tetani E88	NP_781677.1
Clostridium tetani E88	NP_782515.1
Clostridium thermocellum ATCC 27405	YP_001036608.1
Clostridium thermocellum DSM 2360	ZP_05429831.1
Clostridium thermocellum JW20	ZP_06247906.1
Enterococcus casseliflavus ATCC 12755	ZP_08146783.1
Enterococcus casseliflavus EC10	ZP_05653562.1
Enterococcus casseliflavus EC20	ZP_05657320.1
Enterococcus casseliflavus EC30	ZP_05647233.1
Enterococcus faecalis AR01/DG	ZP_05592877.1
Enterococcus faecalis AR01/DG	ZP_05593323.1
Enterococcus faecalis ATCC 29200	ZP_04438493.1
Enterococcus faecalis ATCC 29200	ZP_04438974.1
Enterococcus faecalis ATCC 4200	ZP_05475690.1
Enterococcus faecalis ATCC 4200	ZP_05476148.1
Enterococcus faecalis CH188	ZP_05584030.1
Enterococcus faecalis CH188	ZP_05584487.1
Enterococcus faecalis D6	ZP_05580973.1
Enterococcus faecalis D6	ZP_05581454.1
Enterococcus faecalis DAPTO 512	ZP_07766235.1
Enterococcus faecalis DAPTO 512	ZP_07766769.1
Enterococcus faecalis DAPTO 516	ZP_07768710.1

Enterococcus faecalis DAPTO 516	ZP_07769243.1
Enterococcus faecalis DS5	ZP_05562924.1
Enterococcus faecalis E1Sol	ZP_05576679.1
Enterococcus faecalis Fly1	ZP_05579294.1
Enterococcus faecalis Fly1	ZP_05579836.1
Enterococcus faecalis HH22	ZP_03983099.1
Enterococcus faecalis HH22	ZP_03983671.1
Enterococcus faecalis HIP11704	ZP_05569057.1
Enterococcus faecalis HIP11704	ZP_05569511.1
Enterococcus faecalis JH1	ZP_05573143.1
Enterococcus faecalis JH1	ZP_05573613.1
Enterococcus faecalis Merz96	ZP_05565860.1
Enterococcus faecalis Merz96	ZP_05566313.1
Enterococcus faecalis PC1.1	ZP_06745488.1
Enterococcus faecalis PC1.1	ZP_06745622.1
Enterococcus faecalis R712	ZP_06629084.1
Enterococcus faecalis R712	ZP_06629307.1
Enterococcus faecalis S613	ZP_06632224.1
Enterococcus faecalis S613	ZP_06633438.1
Enterococcus faecalis T1	ZP_05423083.1
Enterococcus faecalis T1	ZP_05423610.1
Enterococcus faecalis T11	ZP_05596079.1
Enterococcus faecalis T11	ZP_05596529.1
Enterococcus faecalis T2	ZP_05426197.1
Enterococcus faecalis T2	ZP_05426649.1
Enterococcus faecalis T3	ZP_05502842.1
Enterococcus faecalis T3	ZP_05503374.1
Enterococcus faecalis T8	ZP_05558277.1
Enterococcus faecalis T8	ZP_05558732.1
Enterococcus faecalis TUSoD Ef11	ZP_07106132.1
Enterococcus faecalis TX0102	ZP_07769831.1
Enterococcus faecalis TX0102	ZP_07771136.1
Enterococcus faecalis TX0104	ZP_03948573.1
Enterococcus faecalis TX0104	ZP_03949050.1
Enterococcus faecalis TX0109	ZP_07568424.1
Enterococcus faecalis TX0109	ZP_07569307.1
Enterococcus faecalis TX0411	ZP_07571129.1
Enterococcus faecalis TX0411	ZP_07571621.1
Enterococcus faecalis TX0470	ZP_07758680.1
Enterococcus faecalis TX0470	ZP_07761048.1
Enterococcus faecalis TX0635	ZP_07762743.1
Enterococcus faecalis TX0635	ZP_07763182.1
Enterococcus faecalis TX0855	ZP_07554224.1
Enterococcus faecalis TX0855	ZP_07554693.1
Enterococcus faecalis TX0860	ZP_07558907.1
Enterococcus faecalis TX0860	ZP_07560137.1
Enterococcus faecalis TX1322	ZP_04434303.1
Enterococcus faecalis TX1322	ZP_04434784.1
Enterococcus faecalis TX2134	ZP_07556868.1
Enterococcus faecalis TX2134	ZP_07558703.1
Enterococcus faecalis TX4248	ZP_07549906.1
Enterococcus faecalis TX4248	ZP_07550773.1
Enterococcus faecalis V583	NP_814987.1
Enterococcus faecalis V583	NP_815511.1

Enterococcus faecalis X98	ZP_05599303.1
Enterococcus faecalis X98	ZP_05599837.1
Enterococcus faecium 1,141,733	ZP_05666270.1
Enterococcus faecium 1,141,733	ZP_05666663.1
Enterococcus faecium 1,230,933	ZP_05658239.1
Enterococcus faecium 1,230,933	ZP_05658868.1
Enterococcus faecium 1,231,408	ZP_05672481.1
Enterococcus faecium 1,231,408	ZP_05674437.1
Enterococcus faecium 1,231,410	ZP_05669697.1
Enterococcus faecium 1,231,410	ZP_05671502.1
Enterococcus faecium 1,231,501	ZP_05663499.1
Enterococcus faecium 1,231,501	ZP_05663934.1
Enterococcus faecium 1,231,502	ZP_05661620.1
Enterococcus faecium 1,231,502	ZP_05662874.1
Enterococcus faecium C68	ZP_05830851.1
Enterococcus faecium C68	ZP_05831097.1
Enterococcus faecium Com12	ZP_05674851.1
Enterococcus faecium Com12	ZP_05675193.1
Enterococcus faecium Com15	ZP_05677472.1
Enterococcus faecium Com15	ZP_05677818.1
Enterococcus faecium D344SRF	ZP_06446591.1
Enterococcus faecium DO	ZP_00603758.1
Enterococcus faecium DO	ZP_00604194.1
Enterococcus faecium DO	ZP_05713383.1
Enterococcus faecium DO	ZP_05714740.1
Enterococcus faecium E1039	ZP_06674694.1
Enterococcus faecium E1039	ZP_06674717.1
Enterococcus faecium E1071	ZP_06678668.1
Enterococcus faecium E1071	ZP_06678810.1
Enterococcus faecium E1162	ZP_06676031.1
Enterococcus faecium E1162	ZP_06677365.1
Enterococcus faecium E1636	ZP_06694171.1
Enterococcus faecium E1636	ZP_06696618.1
Enterococcus faecium E1679	ZP_06699472.1
Enterococcus faecium E1679	ZP_06699604.1
Enterococcus faecium E980	ZP_06681617.1
Enterococcus faecium E980	ZP_06683913.1
Enterococcus faecium PC4.1	ZP_06623476.1
Enterococcus faecium PC4.1	ZP_06624020.1
Enterococcus faecium TC 6	ZP_05921958.1
Enterococcus faecium TX0082	ZP_07851962.1
Enterococcus faecium TX0082	ZP_07852872.1
Enterococcus faecium TX0133A	ZP_07853827.1
Enterococcus faecium TX0133A	ZP_07855510.1
Enterococcus faecium TX0133a01	ZP_07861023.1
Enterococcus faecium TX0133a01	ZP_07861996.1
Enterococcus faecium TX0133a04	ZP_07845782.1
Enterococcus faecium TX0133a04	ZP_07846508.1
Enterococcus faecium TX0133B	ZP_07858484.1
Enterococcus faecium TX0133B	ZP_07859136.1
Enterococcus faecium TX0133C	ZP_07847930.1
Enterococcus faecium TX0133C	ZP_07849791.1
Enterococcus faecium TX1330	ZP_03981917.1
Enterococcus faecium TX1330	ZP_03982263.1

Enterococcus faecium U0317	ZP_06699909.1
Enterococcus faecium U0317	ZP_06702384.1
Enterococcus gallinarum EG2	ZP_05650060.1
Enterococcus italicus DSM 15952	ZP_07894704.1
Exiguobacterium sibiricum 255-15	YP_001813372.1
Exiguobacterium sibiricum 255-15	YP_001815227.1
Exiguobacterium sp. AT1b	YP_002884926.1
Exiguobacterium sp. AT1b	YP_002885951.1
Fructobacillus fructosus KCTC 3544	ZP_08659753.1
Fructobacillus fructosus KCTC 3544	ZP_08660111.1
Geobacillus kaustophilus HTA426	YP_146064.1
Geobacillus sp. C56-T3	YP_003669836.1
Geobacillus sp. G11MC16	ZP_03149251.1
Geobacillus sp. WCH70	YP_002948381.1
Geobacillus sp. Y4.1MC1	YP_003987661.1
Geobacillus sp. Y412MC52	YP_004130846.1
Geobacillus sp. Y412MC61	YP_003252213.1
Geobacillus thermodenitrificans NG80-2	YP_001124318.1
Geobacillus thermoglucosidasius C56-YS93	YP_004586355.1
Lactobacillus acidophilus 30SC	YP_004286780.1
Lactobacillus acidophilus 30SC	YP_004291859.1
Lactobacillus acidophilus ATCC 4796	ZP_04021435.1
Lactobacillus acidophilus ATCC 4796	ZP_04022348.1
Lactobacillus acidophilus NCFM	YP_193368.1
Lactobacillus acidophilus NCFM	YP_193649.1
Lactobacillus amylolyticus DSM 11664	ZP_06818186.1
Lactobacillus amylolyticus DSM 11664	ZP_06818990.1
Lactobacillus amylovorus GRL 1112	YP_004031255.1
Lactobacillus amylovorus GRL 1112	YP_004031563.1
Lactobacillus animalis KCTC 3501	ZP_08548283.1
Lactobacillus animalis KCTC 3501	ZP_08548678.1
Lactobacillus antri DSM 16041	ZP_05745228.1
Lactobacillus antri DSM 16041	ZP_05745282.1
Lactobacillus brevis ATCC 367	YP_795656.1
Lactobacillus brevis ATCC 367	YP_795908.1
Lactobacillus brevis subsp. gravesensis ATCC 27305	ZP_03938709.1
Lactobacillus brevis subsp. gravesensis ATCC 27305	ZP_03940080.1
Lactobacillus buchneri ATCC 11577	ZP_03941690.1
Lactobacillus buchneri ATCC 11577	ZP_03943015.1
Lactobacillus buchneri NRRL B-30929	YP_004397821.1
Lactobacillus buchneri NRRL B-30929	YP_004398936.1
Lactobacillus casei ATCC 334	YP_806117.1
Lactobacillus casei ATCC 334	YP_806356.1
lactobacillus casei BL23	YP_001986885.1
Lactobacillus casei BL23	YP_001987225.1
Lactobacillus casei str. Zhang	YP_003788197.1
Lactobacillus coleohominis 101-4-CHN	ZP_05553087.1
Lactobacillus coleohominis 101-4-CHN	ZP_05553730.1
Lactobacillus coryniformis subsp. coryniformis KCTC 3167	ZP_08476926.1
Lactobacillus coryniformis subsp. coryniformis KCTC 3167	ZP_08477628.1
Lactobacillus coryniformis subsp. coryniformis KCTC 3167	ZP_08478066.1
Lactobacillus coryniformis subsp. torquens KCTC 3535	ZP_08573057.1
Lactobacillus coryniformis subsp. torquens KCTC 3535	ZP_08575297.1
Lactobacillus crispatus 125-2-CHN	ZP_05548315.1

Lactobacillus crispatus 125-2-CHN	ZP_05550241.1
Lactobacillus crispatus 214-1	ZP_06627643.1
Lactobacillus crispatus 214-1	ZP_06627975.1
Lactobacillus crispatus CTV-05	ZP_07789400.1
Lactobacillus crispatus CTV-05	ZP_07789600.1
Lactobacillus crispatus JV-V01	ZP_03995337.1
Lactobacillus crispatus JV-V01	ZP_03997041.1
Lactobacillus crispatus MV-1A-US	ZP_05554243.1
Lactobacillus crispatus MV-1A-US	ZP_05556054.1
Lactobacillus crispatus MV-3A-US	ZP_06018757.1
Lactobacillus crispatus MV-3A-US	ZP_06020877.1
Lactobacillus crispatus ST1	YP_003600919.1
Lactobacillus crispatus ST1	YP_003601225.1
Lactobacillus delbrueckii subsp. bulgaricus ATCC 11842	YP_618758.1
Lactobacillus delbrueckii subsp. bulgaricus ATCC 11842	YP_619549.1
Lactobacillus delbrueckii subsp. bulgaricus ATCC BAA-365	YP_812690.1
Lactobacillus delbrueckii subsp. bulgaricus ATCC BAA-365	YP_813596.1
Lactobacillus delbrueckii subsp. bulgaricus ND02	YP_004033645.1
Lactobacillus delbrueckii subsp. bulgaricus ND02	YP_004034710.1
Lactobacillus delbrueckii subsp. bulgaricus PB2003/044-T3-4	ZP_07091708.1
Lactobacillus delbrueckii subsp. bulgaricus PB2003/044-T3-4	ZP_07092904.1
Lactobacillus farciminis KCTC 3681	ZP_08577352.1
Lactobacillus farciminis KCTC 3681	ZP_08577353.1
Lactobacillus farciminis KCTC 3681	ZP_08577435.1
Lactobacillus fermentum 28-3-CHN	ZP_05863247.1
Lactobacillus fermentum 28-3-CHN	ZP_05863298.1
Lactobacillus fermentum ATCC 14931	ZP_03945340.1
Lactobacillus fermentum ATCC 14931	ZP_03945391.1
Lactobacillus fermentum IFO 3956	YP_001844465.1
Lactobacillus fermentum IFO 3956	YP_001844513.1
Lactobacillus fructivorans KCTC 3543	ZP_08652534.1
Lactobacillus fructivorans KCTC 3543	ZP_08652878.1
Lactobacillus fructivorans KCTC 3543	ZP_08652985.1
Lactobacillus gasseri 202-4	ZP_04644034.1
Lactobacillus gasseri 202-4	ZP_04644299.1
Lactobacillus gasseri 224-1	ZP_06260955.1
Lactobacillus gasseri 224-1	ZP_06261599.1
Lactobacillus gasseri ATCC 33323	YP_815063.1
Lactobacillus gasseri ATCC 33323	YP_815350.1
Lactobacillus gasseri JV-V03	ZP_07057581.1
Lactobacillus gasseri JV-V03	ZP_07059190.1
Lactobacillus gasseri MV-22	ZP_07711627.1
Lactobacillus gasseri MV-22	ZP_07711871.1
Lactobacillus helveticus DPC 4571	YP_001576968.1
Lactobacillus helveticus DSM 20075	ZP_05752798.1
Lactobacillus helveticus DSM 20075	ZP_05753572.1
Lactobacillus hilgardii ATCC 8290	ZP_03953144.1
Lactobacillus hilgardii ATCC 8290	ZP_03953897.1
Lactobacillus iners AB-1	ZP_07267354.1
Lactobacillus iners AB-1	ZP_07267665.1
Lactobacillus iners ATCC 55195	ZP_07906119.1
Lactobacillus iners ATCC 55195	ZP_07906675.1
Lactobacillus iners DSM 13335	ZP_05744512.1
Lactobacillus iners DSM 13335	ZP_05744594.1

Lactobacillus iners LactinV 01V1-a	ZP_07702604.1
Lactobacillus iners LactinV 03V1-b	ZP_07701023.1
Lactobacillus iners LactinV 09V1-c	ZP_07698493.1
Lactobacillus iners LactinV 09V1-c	ZP_07699701.1
Lactobacillus iners LactinV 11V1-d	ZP_07698213.1
Lactobacillus iners LEAF 2052A-d	ZP_07733251.1
Lactobacillus iners LEAF 2052A-d	ZP_07733870.1
Lactobacillus iners LEAF 2053A-b	ZP_07734636.1
Lactobacillus iners LEAF 2053A-b	ZP_07735692.1
Lactobacillus iners LEAF 2062A-h1	ZP_07732070.1
Lactobacillus iners LEAF 2062A-h1	ZP_07732885.1
Lactobacillus iners LEAF 3008A-a	ZP_07731230.1
Lactobacillus iners LEAF 3008A-a	ZP_07731738.1
Lactobacillus iners SPIN 1401G	ZP_08277355.1
Lactobacillus iners SPIN 1401G	ZP_08277654.1
Lactobacillus iners SPIN 2503V10-D	ZP_07703289.1
Lactobacillus iners SPIN 2503V10-D	ZP_07703848.1
Lactobacillus iners UPII 143-D	ZP_08174107.1
Lactobacillus iners UPII 143-D	ZP_08174431.1
Lactobacillus iners UPII 60-B	ZP_08175106.1
Lactobacillus iners UPII 60-B	ZP_08175395.1
Lactobacillus jensenii 115-3-CHN	ZP_05861947.1
Lactobacillus jensenii 115-3-CHN	ZP_05862011.1
Lactobacillus jensenii 1153	ZP_07812304.1
Lactobacillus jensenii 1153	ZP_07812361.1
Lactobacillus jensenii 208-1	ZP_06339483.1
Lactobacillus jensenii 208-1	ZP_06339639.1
Lactobacillus jensenii 208-1	ZP_06339764.1
Lactobacillus jensenii 208-1	ZP_06339995.1
Lactobacillus jensenii 269-3	ZP_04646018.1
Lactobacillus jensenii 269-3	ZP_04646251.1
Lactobacillus jensenii 27-2-CHN	ZP_05556211.1
Lactobacillus jensenii 27-2-CHN	ZP_05556272.1
Lactobacillus jensenii JV-V16	ZP_06923090.1
Lactobacillus jensenii JV-V16	ZP_06923155.1
Lactobacillus jensenii SJ-7A-US	ZP_05866240.1
Lactobacillus jensenii SJ-7A-US	ZP_05866355.1
Lactobacillus johnsonii ATCC 33200	ZP_04007491.1
Lactobacillus johnsonii ATCC 33200	ZP_04008300.1
Lactobacillus johnsonii FI9785	YP_003293415.1
Lactobacillus johnsonii FI9785	YP_003293672.1
Lactobacillus johnsonii NCC 533	NP_965574.1
Lactobacillus kefiranofaciens ZW3	YP_004561944.1
Lactobacillus kefiranofaciens ZW3	YP_004562712.1
Lactobacillus oris PB013-T2-3	ZP_07729573.1
Lactobacillus oris PB013-T2-3	ZP_07730678.1
Lactobacillus paracasei subsp. paracasei 8700:2	ZP_04672241.1
Lactobacillus paracasei subsp. paracasei 8700:2	ZP_04674812.1
Lactobacillus paracasei subsp. paracasei ATCC 25302	ZP_03965452.1
Lactobacillus paracasei subsp. paracasei ATCC 25302	ZP_03965778.1
Lactobacillus plantarum JDM1	YP_003062668.1
Lactobacillus plantarum JDM1	YP_003063658.1
Lactobacillus plantarum subsp. plantarum ATCC 14917	ZP_07077875.1
Lactobacillus plantarum subsp. plantarum ATCC 14917	ZP_07078257.1

Lactobacillus plantarum subsp. plantarum ST-III	YP_003924354.1
Lactobacillus plantarum subsp. plantarum ST-III	YP_003925434.1
Lactobacillus plantarum WCFS1	NP_784935.1
Lactobacillus plantarum WCFS1	NP_786003.1
Lactobacillus reuteri 100-23	ZP_03073097.1
Lactobacillus reuteri 100-23	ZP_03073268.1
Lactobacillus reuteri CF48-3A	ZP_03974394.1
Lactobacillus reuteri CF48-3A	ZP_03975303.1
Lactobacillus reuteri DSM 20016	YP_001272197.1
Lactobacillus reuteri DSM 20016	YP_001272415.1
Lactobacillus reuteri JCM 1112	YP_001842509.1
Lactobacillus reuteri JCM 1112	YP_001842719.1
Lactobacillus reuteri MM2-3	ZP_03848068.1
Lactobacillus reuteri MM2-3	ZP_03848288.1
Lactobacillus reuteri MM4-1A	ZP_08162687.1
Lactobacillus reuteri MM4-1A	ZP_08162912.1
Lactobacillus reuteri SD2112	YP_004649287.1
Lactobacillus reuteri SD2112	YP_004649546.1
Lactobacillus rhamnosus GG	YP_003170576.1
Lactobacillus rhamnosus GG	YP_003170828.1
Lactobacillus rhamnosus HN001	ZP_03211764.1
Lactobacillus rhamnosus HN001	ZP_03212947.1
Lactobacillus rhamnosus Lc 705	YP_003173514.1
Lactobacillus rhamnosus Lc 705	YP_003173849.1
Lactobacillus rhamnosus LMS2-1	ZP_04439526.1
Lactobacillus rhamnosus LMS2-1	ZP_04440656.1
Lactobacillus ruminis ATCC 25644	ZP_08080527.1
Lactobacillus ruminis ATCC 25644	ZP_08081652.1
Lactobacillus ruminis SPM0211	ZP_08563461.1
Lactobacillus ruminis SPM0211	ZP_08563950.1
Lactobacillus sakei subsp. sakei 23K	YP_395078.1
Lactobacillus sakei subsp. sakei 23K	YP_395819.1
Lactobacillus salivarius ACS-116-V-Col5a	ZP_07206764.1
Lactobacillus salivarius ACS-116-V-Col5a	ZP_07207224.1
Lactobacillus salivarius ATCC 11741	ZP_04008335.1
Lactobacillus salivarius ATCC 11741	ZP_04009546.1
Lactobacillus salivarius UCC118	YP_535287.1
Lactobacillus salivarius UCC118	YP_535357.1
Lactobacillus ultunensis DSM 16047	ZP_04011074.1
Lactobacillus ultunensis DSM 16047	ZP_04012165.1
Lactobacillus vaginalis ATCC 49540	ZP_03960087.1
Lactobacillus vaginalis ATCC 49540	ZP_03960157.1
Lactococcus lactis subsp. cremoris MG1363	YP_001033024.1
Lactococcus lactis subsp. cremoris SK11	YP_808837.1
Lactococcus lactis subsp. lactis Il1403	NP_266961.1
Lactococcus lactis subsp. lactis KF147	YP_003353280.1
Leuconostoc argentinum KCTC 3773	ZP_08229369.1
Leuconostoc argentinum KCTC 3773	ZP_08230269.1
Leuconostoc citreum KM20	YP_001727660.1
Leuconostoc citreum KM20	YP_001728953.1
Leuconostoc fallax KCTC 3537	ZP_08312888.1
Leuconostoc fallax KCTC 3537	ZP_08313520.1
Leuconostoc gasicomitatum LMG 18811	YP_003772889.1
Leuconostoc gasicomitatum LMG 18811	YP_003773354.1

Leuconostoc gelidum KCTC 3527	ZP_08478823.1
Leuconostoc gelidum KCTC 3527	ZP_08480233.1
Leuconostoc inhae KCTC 3774	ZP_08482982.1
Leuconostoc kimchii IMSNU 11154	YP_003621634.1
Leuconostoc kimchii IMSNU 11154	YP_003621679.1
Leuconostoc lactis KCTC 3528	ZP_08653839.1
Leuconostoc mesenteroides subsp. cremoris ATCC 19254	ZP_03914617.1
Leuconostoc mesenteroides subsp. cremoris ATCC 19254	ZP_03914695.1
Leuconostoc mesenteroides subsp. mesenteroides ATCC 8293	YP_817569.1
Leuconostoc mesenteroides subsp. mesenteroides ATCC 8293	YP_817886.1
Leuconostoc pseudomesenteroides KCTC 3652	ZP_08656449.1
Leuconostoc pseudomesenteroides KCTC 3652	ZP_08656697.1
Leuconostoc sp. C2	YP_004705937.1
Leuconostoc sp. C2	YP_004705979.1
Listeria grayi DSM 20601	ZP_07054270.1
Listeria innocua Clip11262	NP_470266.1
Listeria ivanovii FSL F6-596	ZP_07873233.1
Listeria marthii FSL S4-120	ZP_07870211.1
Listeria monocytogenes 08-5578	YP_003413122.1
Listeria monocytogenes 08-5923	YP_003416167.1
Listeria monocytogenes 10403S	ZP_05236761.1
Listeria monocytogenes Clip81459	YP_002757653.1
Listeria monocytogenes EGD-e	NP_464452.1
Listeria monocytogenes F6900	ZP_05267635.1
Listeria monocytogenes Finland 1988	ZP_03667815.1
Listeria monocytogenes FSL J1-175	ZP_05387728.1
Listeria monocytogenes FSL J1-194	ZP_05228804.1
Listeria monocytogenes FSL J2-003	ZP_05299120.1
Listeria monocytogenes FSL J2-064	ZP_05275116.1
Listeria monocytogenes FSL J2-071	ZP_06555872.1
Listeria monocytogenes FSL N1-017	ZP_07074131.1
Listeria monocytogenes FSL N3-165	ZP_05232559.1
Listeria monocytogenes FSL R2-503	ZP_05241768.1
Listeria monocytogenes FSL R2-561	ZP_03671412.1
Listeria monocytogenes HCC23	YP_002350651.1
Listeria monocytogenes HPB2262	ZP_05264670.1
Listeria monocytogenes J0161	ZP_05258824.1
Listeria monocytogenes J2818	ZP_05261624.1
Listeria monocytogenes serotype 4b str. F2365	YP_013550.1
Listeria monocytogenes str. 1/2a F6854	ZP_00233812.1
Listeria monocytogenes str. 4b H7858	ZP_00229929.1
Listeria seeligeri serovar 1/2b str. SLCC3954	YP_003464064.1
Listeria welshimeri serovar 6b str. SLCC5334	YP_849104.1
Lysinibacillus fusiformis ZC1	ZP_07048901.1
Lysinibacillus fusiformis ZC1	ZP_07050993.1
Lysinibacillus sphaericus C3-41	YP_001699238.1
Lysinibacillus sphaericus C3-41	YP_001699602.1
Macrococcus caseolyticus JCSC5402	YP_002559872.1
Melissococcus plutonius ATCC 35311	YP_004456432.1
Oceanobacillus iheyensis HTE831	NP_692839.1
Oenococcus oeni ATCC BAA-1163	ZP_01544555.1
Oenococcus oeni AWRIB429	ZP_06552754.1
Oenococcus oeni AWRIB429	ZP_06553215.1
Oenococcus oeni PSU-1	YP_809823.1

Oenococcus oeni PSU-1	YP_810258.1
Paenibacillus polymyxa SC2	YP_003946088.1
Paenibacillus sp. HGF5	ZP_08282147.1
Paenibacillus sp. Y412MC10	YP_003244659.1
Paenibacillus vortex V453	ZP_07901628.1
Pediococcus acidilactici 7_4	ZP_06197007.1
Pediococcus acidilactici 7_4	ZP_06197294.1
Pediococcus acidilactici DSM 20284	ZP_07367696.1
Pediococcus acidilactici DSM 20284	ZP_07368635.1
Pediococcus pentosaceus ATCC 25745	YP_803905.1
Pediococcus pentosaceus ATCC 25745	YP_804133.1
Planococcus donghaensis MPA1U2	ZP_08093775.1
Planococcus donghaensis MPA1U2	ZP_08095321.1
Staphylococcus aureus 930918-3	ZP_06024970.1
Staphylococcus aureus A10102	ZP_06334211.1
Staphylococcus aureus A5937	ZP_05702002.1
Staphylococcus aureus A5948	ZP_05700921.1
Staphylococcus aureus A6224	ZP_05697732.1
Staphylococcus aureus A6300	ZP_05693996.1
Staphylococcus aureus A8115	ZP_05691011.1
Staphylococcus aureus A8117	ZP_06302713.1
Staphylococcus aureus A8796	ZP_06929038.1
Staphylococcus aureus A8819	ZP_06816243.1
Staphylococcus aureus A9299	ZP_05688480.1
Staphylococcus aureus A9635	ZP_05686259.1
Staphylococcus aureus A9719	ZP_05684499.1
Staphylococcus aureus A9754	ZP_06790131.1
Staphylococcus aureus A9763	ZP_05682542.1
Staphylococcus aureus A9765	ZP_06329708.1
Staphylococcus aureus A9781	ZP_05643940.1
Staphylococcus aureus RF122	YP_416162.1
Staphylococcus aureus subsp. aureus 132	ZP_06378138.1
Staphylococcus aureus subsp. aureus 55/2053	ZP_05601265.1
Staphylococcus aureus subsp. aureus 58-424	ZP_06666454.1
Staphylococcus aureus subsp. aureus 65-1322	ZP_05603905.1
Staphylococcus aureus subsp. aureus 68-397	ZP_05606521.1
Staphylococcus aureus subsp. aureus A017934/97	ZP_06375001.1
Staphylococcus aureus subsp. aureus ATCC 51811	ZP_06924986.1
Staphylococcus aureus subsp. aureus ATCC BAA-39	ZP_07364315.1
Staphylococcus aureus subsp. aureus Btn1260	ZP_06312984.1
Staphylococcus aureus subsp. aureus C101	ZP_06331154.1
Staphylococcus aureus subsp. aureus C160	ZP_06311237.1
Staphylococcus aureus subsp. aureus C427	ZP_06326246.1
Staphylococcus aureus subsp. aureus COL	YP_185653.1
Staphylococcus aureus subsp. aureus D139	ZP_06323820.1
Staphylococcus aureus subsp. aureus E1410	ZP_05609199.1
Staphylococcus aureus subsp. aureus ED98	YP_003281607.1
Staphylococcus aureus subsp. aureus EMRSA16	ZP_06819930.1
Staphylococcus aureus subsp. aureus H19	ZP_06342773.1
Staphylococcus aureus subsp. aureus JH1	YP_001315902.1
Staphylococcus aureus subsp. aureus JH9	YP_001246121.1
Staphylococcus aureus subsp. aureus M1015	ZP_06670822.1
Staphylococcus aureus subsp. aureus M809	ZP_06668260.1
Staphylococcus aureus subsp. aureus M876	ZP_05611791.1

Staphylococcus aureus subsp. aureus M899	ZP_06321345.1
Staphylococcus aureus subsp. aureus MN8	ZP_06948993.1
Staphylococcus aureus subsp. aureus MR1	ZP_06857289.1
Staphylococcus aureus subsp. aureus MRSA252	YP_040199.1
Staphylococcus aureus subsp. aureus MSSA476	YP_042812.1
Staphylococcus aureus subsp. aureus Mu3	YP_001441306.1
Staphylococcus aureus subsp. aureus Mu50	NP_371243.1
Staphylococcus aureus subsp. aureus Mu50-omega	ZP_05144111.2
Staphylococcus aureus subsp. aureus MW2	NP_645498.1
Staphylococcus aureus subsp. aureus N315	NP_373929.1
Staphylococcus aureus subsp. aureus NCTC 8325	YP_499287.1
Staphylococcus aureus subsp. aureus str. JKD6009	ZP_03565427.1
Staphylococcus aureus subsp. aureus str. Newman	YP_001331721.1
Staphylococcus aureus subsp. aureus TCH130	ZP_04867002.1
Staphylococcus aureus subsp. aureus TCH70	ZP_07130092.1
Staphylococcus aureus subsp. aureus USA300_FPR3757	YP_493406.1
Staphylococcus aureus subsp. aureus USA300_TCH1516	YP_001574642.1
Staphylococcus aureus subsp. aureus USA300_TCH959	ZP_04865507.1
Staphylococcus aureus subsp. aureus WBG10049	ZP_06318167.1
Staphylococcus aureus subsp. aureus WW2703/97	ZP_06315934.1
Staphylococcus capitis SK14	ZP_03612889.1
Staphylococcus caprae C87	ZP_07840396.1
Staphylococcus carnosus subsp. carnosus TM300	YP_002633465.1
Staphylococcus epidermidis ATCC 12228	NP_764049.1
Staphylococcus epidermidis BCM-HMP0060	ZP_04824622.1
Staphylococcus epidermidis M23864:W1	ZP_04818525.1
Staphylococcus epidermidis M23864:W2(grey)	ZP_06614805.1
Staphylococcus epidermidis RP62A	YP_187973.1
Staphylococcus epidermidis SK135	ZP_06284576.1
Staphylococcus epidermidis W23144	ZP_04796531.1
Staphylococcus haemolyticus JCSC1435	YP_254094.1
Staphylococcus hominis SK119	ZP_04060039.1
Staphylococcus hominis subsp. hominis C80	ZP_07844330.1
Staphylococcus lugdunensis HKU09-01	YP_003472331.1
Staphylococcus lugdunensis M23590	ZP_07911803.1
Staphylococcus pseudintermedius HKU10-03	YP_004148593.1
Staphylococcus saprophyticus subsp. saprophyticus ATCC 15305	YP_302091.1
Staphylococcus warneri L37603	ZP_04677670.1
Streptococcus agalactiae 18RS21	ZP_00779965.1
Streptococcus agalactiae 2603V/R	NP_688379.1
Streptococcus agalactiae 515	ZP_00790860.1
Streptococcus agalactiae A909	YP_330022.1
Streptococcus agalactiae CJB111	ZP_00788426.1
Streptococcus agalactiae COH1	ZP_00784972.1
Streptococcus agalactiae NEM316	NP_735888.1
Streptococcus anginosus 1_2_62CV	ZP_08013217.1
Streptococcus anginosus F0211	ZP_07864423.1
Streptococcus anginosus SK52	ZP_08524858.1
Streptococcus australis ATCC 700641	ZP_08020988.1
Streptococcus bovis ATCC 700338	ZP_07466912.1
Streptococcus cristatus ATCC 51100	ZP_08059350.1
Streptococcus downei F0415	ZP_07725389.1
Streptococcus dysgalactiae subsp. equisimilis GGS_124	YP_002996488.1

Streptococcus equi subsp. equi 4047	YP_002746311.1
Streptococcus equi subsp. zooepidemicus	YP_002744627.1
Streptococcus equi subsp. zooepidemicus MGCS10565	YP_002123234.1
Streptococcus equinus ATCC 9812	ZP_08041305.1
Streptococcus gallolyticus subsp. gallolyticus ATCC BAA-2069	YP_004288263.1
Streptococcus gallolyticus subsp. gallolyticus TX20005	ZP_07464801.1
Streptococcus gallolyticus UCN34	YP_003430779.1
Streptococcus gordonii str. Challis substr. CH1	YP_001450658.1
Streptococcus infantarius subsp. infantarius ATCC BAA-102	ZP_02920791.1
Streptococcus mitis B6	YP_003445868.1
Streptococcus mitis NCTC 12261	ZP_07638926.1
Streptococcus mitis SK321	ZP_07643645.1
Streptococcus mitis SK564	ZP_07645374.1
Streptococcus mitis SK597	ZP_07641938.1
Streptococcus mutans NN2025	YP_003485149.1
Streptococcus mutans UA159	NP_721189.1
Streptococcus parasanguinis ATCC 15912	YP_004621282.1
Streptococcus parasanguinis ATCC 903	ZP_08062854.1
Streptococcus parasanguinis F0405	ZP_07727988.1
Streptococcus parauberis KCTC 11537	YP_004478664.1
Streptococcus parauberis NCFD 2020	ZP_08246484.1
Streptococcus pasteurianus ATCC 43144	YP_004559414.1
Streptococcus porcinus str. Jelinkova 176	ZP_08400045.1
Streptococcus pseudoporcinus SPIN 20026	ZP_07824224.1
Streptococcus pyogenes M1 GAS	NP_269023.1
Streptococcus pyogenes M49 591	ZP_00366447.1
Streptococcus pyogenes MGAS10270	YP_598286.1
Streptococcus pyogenes MGAS10394	YP_059957.1
Streptococcus pyogenes MGAS10750	YP_602203.1
Streptococcus pyogenes MGAS2096	YP_600283.1
Streptococcus pyogenes MGAS315	NP_664345.1
Streptococcus pyogenes MGAS5005	YP_281985.1
Streptococcus pyogenes MGAS6180	YP_280070.1
Streptococcus pyogenes MGAS8232	NP_607024.1
Streptococcus pyogenes MGAS9429	YP_596409.1
Streptococcus pyogenes NZ131	YP_002285648.1
Streptococcus pyogenes SSI-1	NP_802575.1
Streptococcus pyogenes str. Manfredo	YP_001128727.1
Streptococcus salivarius SK126	ZP_04063001.1
Streptococcus sanguinis SK36	YP_001035420.1
Streptococcus sanguinis VMC66	ZP_08086950.1
Streptococcus sp. 2_1_36FAA	ZP_06060719.1
Streptococcus sp. C150	ZP_08047096.1
Streptococcus sp. M334	ZP_08050744.1
Streptococcus sp. oral taxon 056 str. F0418	ZP_08662480.1
Streptococcus suis 05HAS68	ZP_07249220.1
Streptococcus suis 05ZYH33	YP_001198632.1
Streptococcus suis 89/1591	ZP_03626014.1
Streptococcus suis 98HAH33	YP_001200838.1
Streptococcus suis BM407	YP_003028445.1
Streptococcus suis P1/7	YP_003026960.1
Streptococcus suis SC84	YP_003025135.1
Streptococcus suis ST3	YP_004401360.1

Streptococcus thermophilus CNRZ1066	YP_141047.1
Streptococcus thermophilus LMD-9	YP_820140.1
Streptococcus thermophilus LMG 18311	YP_139157.1
Streptococcus uberis 0140J	YP_002562053.1
Streptococcus vestibularis ATCC 49124	ZP_08070018.1
Streptococcus vestibularis F0396	ZP_07723832.1
Syntrophobotulus glycolicus DSM 8271	YP_004265232.1
Thermanaerovibrio acidaminovorans DSM 6589	YP_003316888.1
Thermincola sp. JR	YP_003640170.1
Thermotoga thermarum DSM 5069	YP_004659638.1
Weissella cibaria KACC 11862	ZP_08416118.1
Weissella cibaria KACC 11862	ZP_08417007.1
Weissella koreensis KACC 15510	YP_004725936.1
Weissella koreensis KACC 15510	YP_004726330.1
Weissella paramesenteroides ATCC 33313	ZP_04782029.1
Weissella paramesenteroides ATCC 33313	ZP_04782820.1
